# Effect of Substituents with the Different Electron-Donating Abilities on Optoelectronic Properties of Bipolar Thioxanthone Derivatives

**DOI:** 10.1021/acsaelm.3c00092

**Published:** 2023-03-28

**Authors:** Simas Macionis, Dalius Gudeika, Dmytro Volyniuk, Malek Mahmoudi, Jurate Simokaitiene, Viktorija Andruleviciene, Murad Najafov, Rita Sadzeviciene, Sigitas Stoncius, Juozas V. Grazulevicius

**Affiliations:** † Department of Polymer Chemistry and Technology, Kaunas University of Technology, K. Barsausko st. 59, LT-51423 Kaunas, Lithuania; ‡ Department of Organic Chemistry, Center for Physical Sciences and Technology, Sauletekio Ave. 3, LT-10257 Vilnius, Lithuania

**Keywords:** thioxanthone, carbazole, phenothiazine, acridane, solid-state luminescence enhancement, thermally activated delayed fluorescence, electroluminescence, organic light-emitting diode

## Abstract

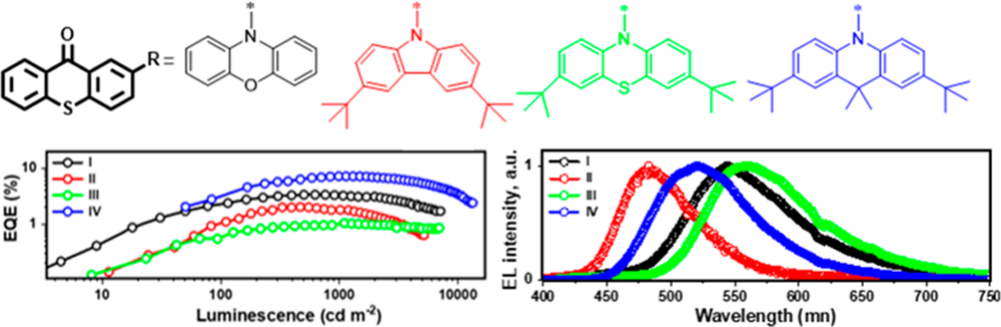

The synthesis and optoelectronic properties of four simple-structure thioxanthone derivatives employing thioxanthone as an acceptor unit, coupled with moieties having very different electron-donating abilities such as phenoxazine, 3,6-di-*tert*-butylcarbazole, 3,7-di-*tert-*butylphenothiazine, or 2,7-di-*tert*-butyl-9,9-dimethylacridane, are reported. The compounds form molecular glasses with glass transition temperatures reaching 116 °C. Ionization potentials of the compounds estimated by photoelectron emission method range from 5.42 to 5.74 eV. Thioxanthone derivatives containing 3,6-*tert*-butylcarbazole or 2,7-di-*tert*-butyl-9,9-dimethylacridane moieties with weak electron-donating strengths were characterized by bipolar charge transport with relatively close hole and electron mobility values of 6.8 × 10^–5^/2.4 × 10^–5^ and 3.1 × 10^–5^/4.6 × 10^–6^ cm^2^/(V s) recorded at 3.6 × 10^5^ V/cm. The other compounds demonstrated hole-transporting properties. The films of thioxanthones containing phenoxazine or 2,7-di-*tert*-butyl-9,9-dimethylacridane moieties showed efficient thermally activated delayed fluorescence with a photoluminescence quantum yield of up to 50% due to the solid-state luminescence enhancement. Organic-light-emitting diodes containing the synthesized compounds as emitters showed very different external quantum efficiencies (0.9–10.3%) and blue, sky blue, green, or yellow electroluminescence colors, thus reflecting the effects of donor substituents.

## 1 Introduction

For more than three decades, organic light-emitting diodes (OLEDs) have been widely studied by the scientific community of organic electronics, due to their potentially low cost, low weight, rapid response, flexibility, and other advantages.^1^ The design of new organic semiconductors with desirable characteristics is essential with respect to the performance, lifetime, and advancement of OLED displays and lighting devices. Metal-atom-free donor–acceptor (D-A) type semiconductors are widely used in light-emitting layers of OLEDs. The required properties for efficient electroluminescence are as follows:

- bipolar charge-transporting properties with balanced hole and electron mobilities^2^
- hole and electron injection abilities with low energy barriers for charge carriers^3^
- high thermal stabilities and high glass transition temperatures^4^
- high photoluminescence quantum yields (PLQYs) of the solid films which can be achieved due to solid-state luminescence enhancement (SSLE) or so-called aggregation-induced emission enhancement (AIEE)^5^
- triplet harvesting via thermally activated delayed fluorescence (TADF),^6^ room-temperature phosphorescence (RTP),^7^ triplet–triplet annihilation (TTA),^8^ etc.

Aiming to obtain organic semiconductors with the desirable combination of optoelectronic properties, a number of donor–acceptor compounds containing thioxanthone as the acceptor moiety were synthesized. The solutions of carbazolyl-containing thioxanthones in methylene chloride were characterized by blue prompt fluorescence (PF) peaking at 443 nm with PLQYs of 84–85%.^9^ The molecular dispersions of derivatives of thioxanthone and carbazole in polystyrene demonstrate RTP under an inert atmosphere.^10^ TTA properties were detected for a family of thioxanthone derivatives containing nitrophenyl or benzoyl groups.^11^ In contrast, unsubstituted thioxanthone showed triplet harvesting abilities via upper triplet–singlet reverse intersystem crossing (RISC), which is promising for OLED applications.^12^ Inefficient blue TADF was observed for fluorenyl-disubstituted thioxanthone.^13^ This compound was used as the TADF host for red phosphorescent emitters in OLEDs which showed a maximum external quantum efficiency (EQE) of 10.4% and low-efficiency rolloff. Efficient blue, yellow, and white OLEDs with maximum EQEs of up to 23.7% were fabricated using derivatives of thionylamine and thioxanthone as emitters.^14^ The simple donor–acceptor (D-A) derivative of carbazole and thioxanthone showed blue TADF, allowing the fabrication of OLEDs with a maximum EQE of 11.2%.^15^ Additional modifications of molecular structures of derivatives of carbazole and thioxanthone allowed reaching maximum EQEs of TADF OLEDs of 24.4%.^15−17^ Thus, a thioxanthone moiety is a valuable building block for the design of D-A type TADF emitters.^18−21^ However, the reported D-A-type thioxanthone derivatives were designed using limited types of donors, ignoring many other interesting donors used for the development of TADF materials. We partly aimed to investigate the effect of donor substituents with different electron-donating strengths on the optoelectronic properties of bipolar thioxanthone derivatives with a simple D-A molecular structure using a wide set of experimental approaches.

**Scheme 1:**
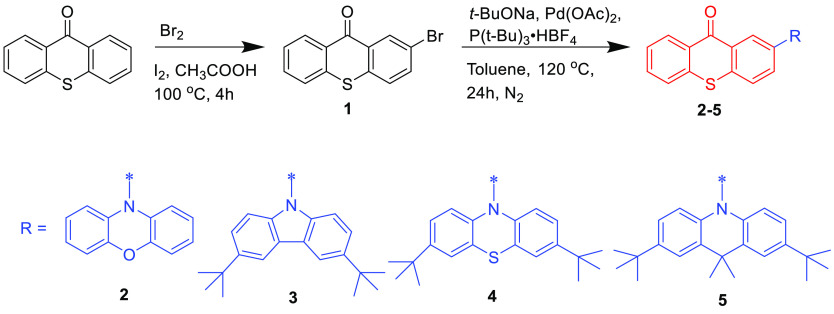
Synthesis of Compounds **2**–**5**

In this work, we report on a series of thioxanthone D-A-type derivatives containing different donor moieties (phenoxazine, 3,6-di-*tert*-butylcarbazole, 3,7-di-*tert*-butylphenothiazine, or 2,7-di-*tert*-butyl-9,9-dimethylacridane) (Scheme 1.). The impact of donor substituents with different electron-donating strengths on the photophysical, electrochemical, and photoelectrical properties of the thioxanthone derivatives is discussed. Two compounds are characterized by a small singlet–triplet energy gap (Δ*E*_ST_), which are necessary for TADF effect. Three compounds demonstrate an SSLE effect, causing a relatively high PLQY (up to 50%) of the solid samples. The compounds were characterized by relatively good hole- and electron-injecting properties, high thermal stability, and high glass transition temperatures reaching 116 °C. Bipolar charge-transporting properties were proved for two compounds by time-of-flight measurements. Due to the different effects of donor substituents on electroluminescent properties, OLEDs based on the differently substituted thioxanthones were characterized by blue, sky blue, green, and yellow electroluminescence with different efficiencies.

## 2 Experimental Section

### 2.1 General Considerations

^1^H NMR and ^13^C NMR spectra were recorded using a Varian Unity Inova spectrometer (300 MHz (^1^H), 75.4 MHz (^13^C)). Infrared (IR) and mass (MS) spectra, elemental and thermogravimetric analysis, and DSC measurements were carried out as described previously.^22^ An electrothermal MEL-TEMP apparatus was used for the estimation of melting points. Cyclic voltammetry (CV) measurements were carried out as described previously.^23^ To record PL and phosphorescence spectra, as well as PL decay curves at different temperatures, a liquid nitrogen cryostat (Optistat DN2) was used. For measurements of the dependences of delayed emission intensity on the laser flux of the samples, an Edinburgh Instruments FLS980 spectrometer and a PicoQuantLDH-D-C-375 laser (wavelength 374 nm) were utilized. Ionization potential measurements of solid samples were performed by the photoelectron emission method in air. Charge mobility values were studied by the time-of-flight (TOF) method. Various positive and negative external voltages (*U*) were applied to the samples using a 6517B electrometer (Keithley) to check hole and electron transport in the layers at the different electric fields. A TDS 3032C oscilloscope (Tektronix) was used to record the photocurrent transients of holes or electrons. Charge mobilities were estimated by the formula μ = *d*^2^/(*U × t*_tr_), where *t*_tr_ is the transit time, *d* is the thickness of a layer, and *U* is the applied voltage over the sample.

### 2.2 Device Fabrication and Measurements

The functional materials used for OLED fabrication were purchased from Merck and Ossila and used as received without further purification. OLEDs were fabricated by a vacuum thermal evaporation method. Iodide tin oxide (ITO) substrates were prepared with 5 min of UV–ozone cleaner. The cleaned substrates were immediately transferred to a glovebox with an inbuilt evaporator system vacuum chamber. The deposition rate was controlled by an SQC 310 controller with two quartz crystal sensors. The deposition rate of the organic layers was 0.8–1.2 Å/s, while the aluminum deposition rate was 1.5 Å/s. The electroluminescence and current density–voltage characteristics were determined using the certificated photodiode PH100-Si-HA-D0 together with a PC-Based Power and Energy Monitor 11S-LINK (from STANDA) and Keithley 2400C source meter. An Avantes AvaSpec-2048XL spectrometer was used to take electroluminescence (EL) spectra. Device efficiencies were calculated using the luminance, current density, and EL spectra.

### 2.3 Materials

10*H*-Phenoxazine, palladium(II) acetate (Pd(OAc)_2_), P(*t*-Bu)_3_·HBF_4_, and sodium *tert*-butoxide (*t*-BuONa) as well as the solvents used were purchased from Aldrich and used as received. Thioxanthone derivatives **2**–**5** were synthesized by Buchwald–Hartwig coupling reactions from 2-bromo-9*H*-thioxanthen-9-one and the respective donor. Molecular structures of the synthesized thioxanthones with the corresponding donor moieties phenoxazine, 3,6-di-*tert*-butyl-9*H*-carbazole, 3,7-di-*tert*-butyl-10*H*-phenothiazine, and 2,7-di-*tert*-butyl-9,9-dimethyl-9,10-dihydroacridine are shown in Scheme 1. Their chemical structures were confirmed by ^1^H and ^13^C NMR, IR, and mass spectroscopy. All the compounds showed good solubility in common organic solvents (THF, chloroform, acetone, etc.). The intermediate compound 2-bromo-9*H*-thioxanthen-9-one (**1**) was prepared by bromination of 9*H*-thioxanthen-9-one with bromine and iodine in acetic acid, according to the previously described procedure.^24^ 3,6-Di-*tert*-butyl-9*H*-carbazole, 3,7-di-*tert*-butyl-10*H*-phenothiazine, and 2,7-di-*tert*-butyl-9,9-dimethyl-9,10-dihydroacridine were synthesized according to the reported procedures.^25,26^

#### 2.3.1 2-(10*H*-Phenoxazin-10-yl)-9*H*-thioxanthen-9-one (**2**)

2-Bromo-9*H*-thioxanthen-9-one (0.5 g, 1.17 mmol) and 10*H*-phenoxazine (0.36 g, 1.97 mmol) were dissolved in 9 mL of toluene, *t*-BuONa (0.41 g, 4.29 mmol), Pd(OAc)_2_ (0.0071 g, 0.034 mmol), and P(*t*-Bu)_3_·HBF_4_ (0.05 g, 0.17 mmol) were added, the temperature of the reaction mixture was increased to 120 °C, and the reaction mixture was stirred for 24 h under nitrogen. The reaction was monitored by TLC. When it was finished, the reaction mixture was poured into ice-cold water, the crude product was extracted with dichloromethane, and the organic fraction was dried with Na_2_SO_4_. The target product was purified using column chromatography (eluent toluene) and then recrystallized from methanol/chloroform to afford 0.3 g (44%) of the target product as yellow crystals. Mp: 252–253 °C. ^1^H NMR (400 MHz, CDCl_3_, δ, ppm): 8.56 (d, *J* = 8.6 Hz, 2H), 7.75 (d, *J* = 8.6 Hz, 1H), 7.64–7.51 (m, 3H), 7.46 (t, *J* = 7.8 Hz, 1H), 6.65 (d, *J* = 7.5 Hz, 2H), 6.60 (t, *J* = 7.1 Hz, 2H), 6.51 (d, *J* = 7.7 Hz, 2H), 5.87 (d, *J* = 7.8 Hz, 2H). ^13^C NMR (75 MHz, CDCl_3_, δ, ppm): 179.1, 144.0, 137.4, 136.8, 135.1, 133.9, 132.8, 132.6, 131.5, 130.1, 129.2, 128.8, 126.8, 126.1, 123.3, 121.7, 115.6, 113.3. IR (in KBr), cm^–1^: 3063 ν(CH_ar_); 2889, 2873 ν(CH_aliphatic_); 1632 ν(C=O_xanthone_); 1592, 1489, 1458 ν(C=C_ar_); 1337, 1292, 1273 ν(C–N); 860, 825, 737 γ(CH_ar_). MS (APCI^+^, 20 V), *m*/*z*: 393 ([M]^+^).

#### 2.3.2 2-(3,6-Di-*tert*-butyl-9*H*-carbazol-9-yl)-9*H*-thioxanthen-9-one (**3**)

2-Bromo-9*H*-thioxanthen-9-one (0.5 g, 1.17 mmol) and 3,6-di-*tert*-butyl-9*H*-carbazole (0.55 g, 1.97 mmol) were dissolved in 9 mL of toluene, *t*-BuONa (0.41 g, 4.29 mmol), Pd(OAc)_2_ (0.0071 g, 0.034 mmol), and P(*t*-Bu)_3_·HBF_4_ (0.05 g, 0.17 mmol) were added, the temperature of the reaction mixture was increased to 120 °C, and the reaction mixture was stirred for 24 h under nitrogen. The reaction was monitored by TLC. When it was finished, the reaction mixture was poured into ice-cold water, and the crude product was extracted with dichloromethane and dried with Na_2_SO_4_. The target product was purified using column chromatography (eluent toluene) and then recrystallized from methanol/chloroform to afford 0.41 g (49%) of the target product as yellow crystals. Mp: 251–252 °C. ^1^H NMR (400 MHz, CDCl_3_, δ, ppm): 8.77 (d, *J* = 2.1 Hz, 1H), 8.57 (d, *J* = 8.2 Hz, 1H), 8.08 (s, 2H), 7.80–7.53 (m, 4H), 7.48–7,35 (m, 3H), 7.32 (d, *J* = 8.7 Hz, 2H), 1.40 (s, 18H). ^13^C NMR (75 MHz, CDCl_3_, δ, ppm): 179.4, 143.4, 138.9, 137.1, 132.5, 130.6, 130.1, 127.6, 127.1, 126.6, 126.1, 123.8, 123.6, 116.3, 106.0, 34.7, 32.0. IR (in KBr), cm^–1^: 3046 ν(CH_ar_); 2896, 2861 ν(CH_aliphatic_); 1638 ν(C=O_xanthone_); 1594, 1485, 1436 ν(C=C_ar_); 1363, 1296, 1264 ν(C–N); 893, 809, 742 γ(CH_ar_). MS (APCI^+^, 20 V), *m*/*z*: 491 ([M + H]^+^).

#### 2.6 2-(3,7-Di-*tert*-butyl-10*H*-phenothiazin-10-yl)-9*H*-thioxanthen-9-one (**4**)

2-Bromo-9*H*-thioxanthen-9-one (0.5 g, 1.17 mmol) and 3,7-di-*tert*-butyl-10*H*-phenothiazine (0.61 g, 1.97 mmol) were dissolved in 9 mL of toluene, *t*-BuONa (0.41 g, 4.29 mmol), Pd(OAc)_2_ (0.0071 g, 0.034 mmol), and P(*t*-Bu)_3_·HBF_4_ (0.05 g, 0.17 mmol) were added, the temperature of the reaction mixture was increased to 120 °C, and the reaction mixture was stirred for 24 h under nitrogen. The reaction was monitored by TLC. When it was finished, the reaction mixture was poured into ice-cold water and the crude product was extracted with dichloromethane and dried with Na_2_SO_4_. The target product was purified using column chromatography (eluent toluene) and then recrystallized from methanol/chloroform to afford 0.35 g (39%) of the target product as yellow crystals. Mp: 201–202 °C. ^1^H NMR (400 MHz, CDCl_3_, δ, ppm): 8.65 (d, *J*_1_ = 8.6 Hz, 2H), 7.74 (d, *J* = 8.6 Hz, 1H), 7.71–7.59 (m, 3H), 7.57–7.49 (m, 1H), 7.17 (d, *J* = 2.2 Hz, 2H), 6.97 (dd, *J*_1_ = 8.6 Hz, *J*_2_ = 2.3 Hz, 2H), 6.45 (d, *J* = 8.6 Hz, 2H), 1.28 (s, 18H). ^13^C NMR (75 MHz, CDCl_3_, δ, ppm): 179.3, 146.5, 141.1, 137.1, 132.2, 130.8, 130.0, 128.2, 126.5, 126.1, 124.4, 123.9, 123.2, 118.2, 34.1, 31.2. IR, (in KBr), cm^–1^: 3039 ν(CH_ar_); 2892, 2858 ν(CH_aliphatic_); 1631 ν(C=O_xanthone_); 1589, 1487, 1426 ν(C=C_ar_); 1372, 1288, 1258 ν(C–N); 887, 803, 739 γ(CH_ar_). MS (APCI^+^, 20 V), *m*/*z*: 522 ([M + H]^+^).

#### 2.7 2-(2,7-Di-*tert*-butyl-9,9-dimethylacridin-10(9*H*)-yl)-9*H*-thioxanthen-9-one (**5**)

2-Bromo-9*H*-thioxanthen-9-one (0.5 g, 1.17 mmol) and 2,7-di-*tert*-butyl-9,9-dimethyl-9,10-dihydroacridine (0.63 g, 1.97 mmol) were dissolved in 9 mL of toluene, *t*-BuONa (0.41 g, 4.29 mmol), Pd(OAc)_2_ (0.0071 g, 0.034 mmol), and P(*t*-Bu)_3_·HBF_4_ (0.05 g, 0.17 mmol) were added, the temperature of the reaction mixture was increased to 120 °C, and the reaction mixture was stirred for 24 h under nitrogen. The reaction was monitored by TLC. When it was finished, the reaction mixture was poured into ice-cold water, and the crude product was extracted with dichloromethane and dried with Na_2_SO_4_. The target product was purified using column chromatography (eluent toluene) and then recrystallized from methanol/chloroform to afford 0.38 g (42%) of the target product as yellow crystals. Mp: 238–239 °C. ^1^H NMR (400 MHz, CDCl_3_): δ 8.56 (d, *J* = 2.7 Hz, 2H), 7.75 (d, *J* = 8.6 Hz, 1H), 7.61–7.50 (m, 3H), 7.47–7.40 (m, 3H), 6.89 (dd, *J*_1_ = 8.6 Hz, *J*_2_ = 2.7 Hz, 2H), 6.11 (d, *J* = 8.6 Hz, 2H), 1.67 (s, 6H), 1.23 (s, 18H). ^13^C NMR (75 MHz, CDCl_3_, δ, ppm): 179.3, 143.2, 139.9, 138.5, 136.9, 136.8, 135.9, 132.9, 132.5, 131.5, 130.1, 129.7, 128.9, 128.6, 126.6, 126.1, 123.1, 122.2, 113.4, 34.2, 31.5. IR (in KBr), cm^–1^: 3054 ν(CH_ar_); 2963, 2901, 2865 ν(CH_aliphatic_); 1639 ν(C=O_xanthone_); 1593, 1491, 1438 ν(C=C_ar_); 1362, 1333, 1283 ν(C–N); 893, 808, 748 γ(CH_ar_). MS (APCI^+^, 20 V), *m*/*z*: 533 ([M + H]^+^).

## 3 Results and Discussion

### 3.1 Photophysical Properties

**Figure 1:**
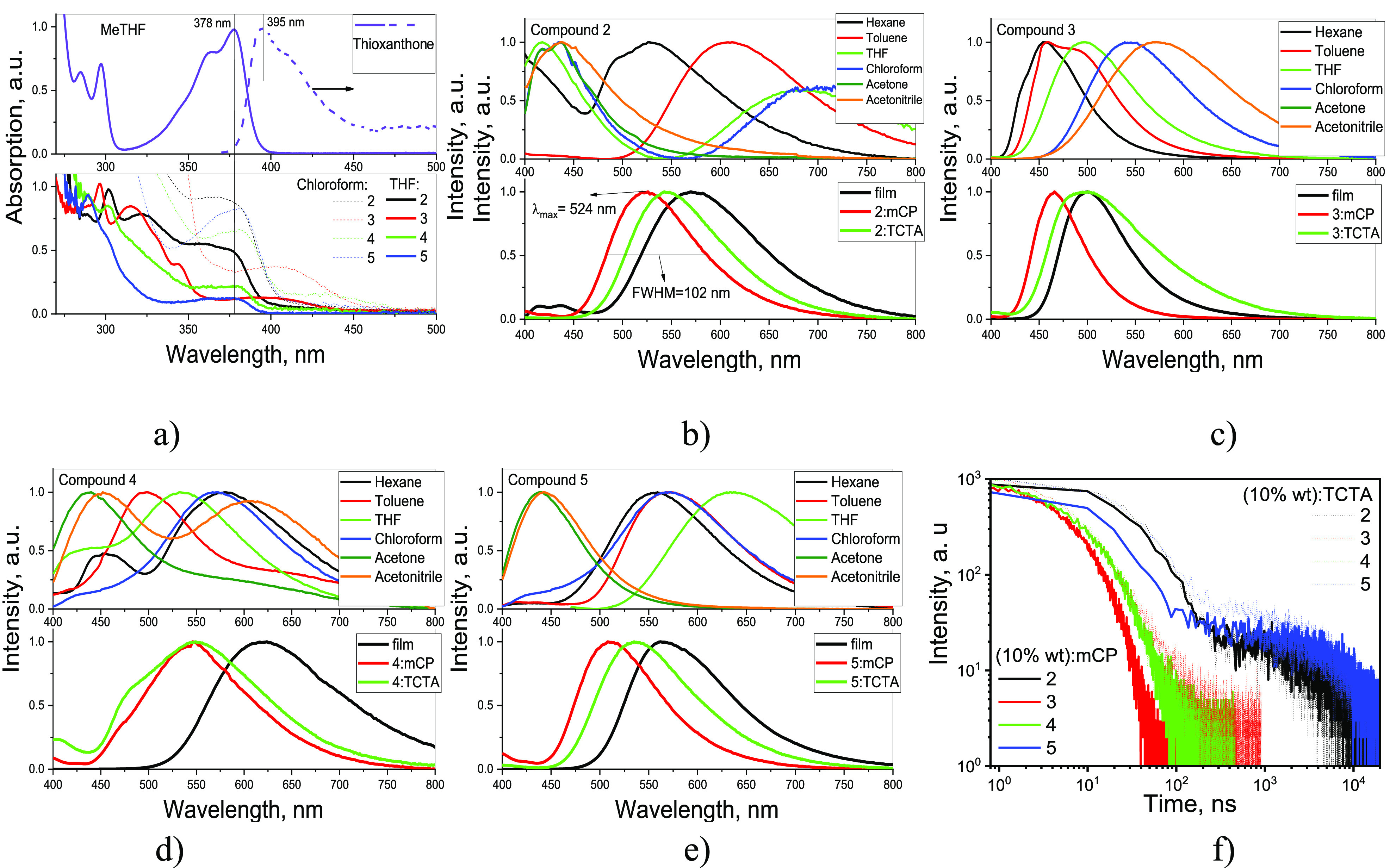
Absorption spectra (a) of the solutions (10^–5^ M) of thiaxanthone and compounds **2**–**5**. PL spectra (b–e) of the solutions (10^–5^ M), thin films of compounds **2**–**5**, and thin films of compounds **2**–**5** (10 wt %) doped in mCP or TCTA (λex = 330 nm). PL decay curves (f) of thin films of **2**–**5** doped in mCP or TCTA (λex = 374 nm, λem = λmax nm).

To investigate the electronic structure of thioxanthone derivatives in the ground state, absorption spectra of the THF and chloroform solutions of compounds **2**–**5** were recorded. They are shown in Figure 1a. For a comparison, the absorption spectrum of an Me-THF solution of thioxanthone is plotted in Figure 1a. The low-energy bands (tails) observed at wavelengths longer than 390 nm are the signs of the formation of the states of charge transfer (CT) from one of the donor moieties to the thioxanthone unit. Taking into account the most intense absorption of the CT stats of compound **3**, it can be presumed that the most efficient charge transfer is observed between 3,6-di-*tert*-butylcarbazole and thioxanthone fragments. An efficient charge transfer was also observed between the 3,7-di-*tert-*butylphenothiazine and thioxanthone units of compound **4**. Meanwhile, very weak absorption of the CT states was observed for the solutions of compounds **2** and **5**. It was manifested in the low-energy tails in the absorption spectra of their solutions (Figure 1a). Similar to the solutions of all the compounds (**2**–**5**), absorption bands observed at ca. 380 nm belong to π–π * transitions of the thioxanthone units. These bands are slightly bathochromicaly shifted in comparison to the corresponding band of thioxanthone. This observation can be attributed to the extended conjugation of compounds **2**–**5**. The different shapes of absorption spectra of the solutions of **2**–**5** were observed in the range of 300–360 nm. The absorption in this range results from π–π* transitions of the different electron-donating units: i.e., phenoxazine, 3,6-di-*tert*-butylcarbazole, 3,7-di-*tert-*butylphenothiazine, or 2,7-di-*tert*-butyl-9,9-dimethylacridane. Thus, the absorption spectra of the solutions of **2**–**5** at wavelengths lower than 380 nm mainly resulted from the sum of the absorptions of donor and acceptor units. Meanwhile, the contributions of CT states in the ground state are detectable at wavelengths higher than 400 nm. The absorption spectra of the solutions of **2**–**5** practically did not show a solvatochromic effect. However, considerable solvatochromic effects were reflected in the photoluminescence spectra of the solutions of the compounds (Figure 1b–e).

The effect of polarity of the solvents on the PL spectra of the solutions of the compounds was studied (Figure 1b–e). Double emission was detected for the solutions of compounds **2**–**5** in the different solvents. The PL spectrum of the solution of compound **3** in hexane demonstrated a slightly structured PL band (Figure 1c). Time-dependent density functional theory (TD-DFT) (Figure S1) revealed that the first excited singlet state (S_1_) of compound **3** can be attributed to the combination of the local excitation (LE) in the 3,6-*tert*-butylcarbazole fragment and CT between carbazole and thioxanthone moieties. However, the calculated S_2_ of compound **3** shows pure CT between donor and acceptor moieties. The PL spectra of the solutions of compound **3** have a single band, the wavelength of the maximum of which increases with an increase in dielectric constants of the solvents (Figure 1c). The emission of compound **3** is related to the emissive relaxations of CT states. This observation indicates that S_2_ became emissive because of its sensitivity to the polarity of the solvents. The interplay between S_1_ and S_2_ confirms the PL spectrum of the solution in toluene, which shows double emission of both excited states. Some PL spectra of the solutions of compounds **2**, **4**, and **5** were characterized by two high-energy and low-energy bands. TD-DFT calculations showed that the difference between S_1_ and S_2_ of these compounds in the gas phase ranged from 0.65 to 0.92 eV (Figure S1). The photophysical properties of donor–acceptor type compounds **2**, **4**, and **5** are sensitive to the polarity of the solvents; therefore, the gap between S_1_ and S_2_ can increase for the solutions in more polar solvents ,resulting in double emission or emission from a higher excited state. The possible emissions from S_1_ and/or S_2_ of the solutions of compounds **2**, **4**, and **5** in the different solvents are presented in Figure S1. The low-energy band of the PL spectrum of the solution of compound **2** in hexane was observed at 529 nm, while for the solution in chloroform, it was recorded at 703 nm. The high-energy bands of PL spectra of the different solutions of compound **2** were observed in the range of ca. 390–470 nm. The wavelengths (ca. 418 and 438 nm) of the maxima of that band were practically insensitive to the polarity of the solvents. Rather, the slight shifts of the high-energy PL peaks are related to the overlapping of LE and CT emissions.

Similar solvatochromic properties were observed for PL spectra of the solutions of compounds **4** and **5** (Figure 1d,e). Two bands can be recognized in PL spectra of the solutions of compounds **4** and **5**: i.e., the LE band at ca. 340 nm and CT band at wavelengths longer than 530 nm. For an investigation of the effects of conformational and static dielectric disorders on the photophysical properties of compounds **2**–**5** in the solid state, PL spectra of neat films of **2**–**5** and of the films of compounds **2**–**5** (10 wt %) doped in hosts with different dielectric constants (ε), i.e., 1,3-bis(*N*-carbazolyl)benzene (*m*CP) with ε = 2.84^27^ or tris(4-carbazoyl-9-ylphenyl)amine (TCTA) with ε = 5.61,^27^ were recorded (Figure 1b–e).

PL spectra of the solid films of compounds **2**–**5** doped in mCP and in TCTA as well as of the films of neat compounds (**2**–**5**) are characterized by single bands with different full widths at half-maxima (fwhm) and the different wavelengths of PL maxima (Figure 1b–e). For example, fwhm values of 102, 110, and 134 nm and λ_max_ values of 524, 545, and 573 nm were observed for the films of compound **2** doped with mCP and in TCTA and of neat compound **2**, respectively. Similar trends were observed for the solid samples of other studied compounds, reflecting the effects of conformational and static dielectric disorders on the photophysical properties. In contrast to PL spectra of the solutions, only weak contributions of LE emission at wavelengths shorter than 450 nm were detected for the solid samples of compounds **2**–**5**.

As was theoretically predicted,^28^ PL spectra and PL lifetimes of donor–acceptor type compounds can be widely varied due to the conformational and static dielectric disorder effects. Molecular dispersions of compounds **2** and **5** demonstrated prompt (PF) and delayed (DF) fluorescence, while those of compounds **3** and **4** showed only PF. PL spectra and PL lifetimes of the solid samples of compounds **2**–**5** can apparently be widely varied due to the flexible linkage of the donor and acceptor with only limited steric restrictions of movements of the molecular fragments (vibrations or rotations). This presumption is in good agreement with the low PLQY values of the solutions of the compounds (Table 1). The PLQY values of the films of compounds **2**, **3**, and **5** were found to be higher than those of the solutions because of the decrease of nonemissive energy losses due to the restriction of molecular vibrations or rotations. Such an observation is typical for luminophores exhibiting aggregation-induced emission enhancement (AIEE).^5^ However, the highest PLQY values were observed for the films of the compounds doped in mCP, which is widely used as a host of emitting layers of OLEDs. They ranged from 23 to 50%. The highest PLQY was observed for the film of compound **5** doped in mCP. We consider that the enhanced emission intensity of compounds **2** and **5** in the solid state can be attributed to their TADF properties. Because of the conformational and static dielectric disorder effects,^27^ TADF of neat films is typically inefficient. As a result, PLQYs of the neat films of compounds **2** and **5** are low (Table 1). The use of hosts allows increasing the TADF efficiency of organic compounds and achieving higher PLQY values (Table 1).^29,30^

**Table 1:** Wavelengths of Emission Maxima and PLQY Values of the Solutions and Solid Samples of Compounds **2**–**5**

		compound			
	medium	**2**	**3**	**4**	**5**
λ_max_, nm	toluene/neat	606/573	457, 492*/502	498, 652*/620	570/568
	mCP/TCTA	524/545	465/493	546, 479*/546, 479*	509/536
fwhm, nm	mCP	102	59	148	95
PLQY, %	toluene/neat/mCP	1/7/36	2/5/24	5/3/23	1/18/50
Δ*E*_ST_, eV	mCP	0.07	0.37	0.16	0.11

**Figure 2:**
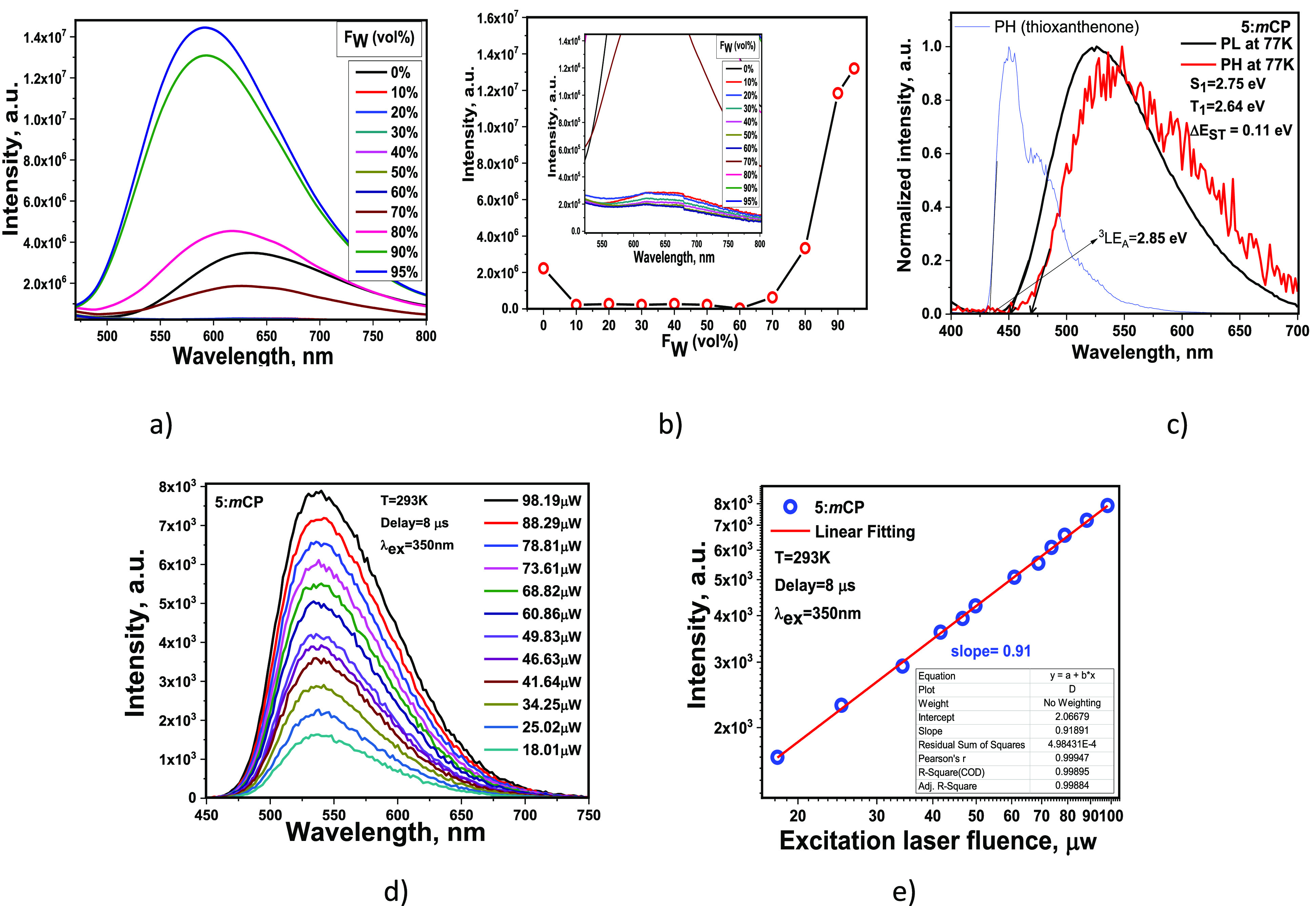
(a) PL spectra of the dispersions of compound **5** in a mixtures of THF and water of various water fractions. (b) Plot of PL maximum intensity versus water volume fraction for the dispersions of compound **5** in THF/water mixtures. (c) PL and PH spectra of compound **5** doped in an *m*CP and MeTHF solution of thioxanthone recorded at 77 K. PL spectra (d) recorded at different excitation powers and delayed fluorescence intensity versus excitation power (e) for the film of compound **5** doped in *m*CP. PH spectra were recorded with a delay of 1 ms after excitation.

The effect of AIEE was studied in more detail using dispersions of compound **5** in the mixtures of THF and water (Figure 2a,b). CT emission at 662 nm was practically not detectable for the dispersion of compound **5** in water/THF mixtures at water fractions (*f*_w_) of 10–60% without enlargement (Figure 2b, inset). At *f*_w_ higher than 60%, aggregates started to be formed due to the poor solubility of **5** in the mixtures of the solvents. The dispersion of compound **5** exhibited clear CT emission at 626 nm at *f*_w_ of 70%. The PL intensity increased considerably at *f*_w_ of 80% and further increased up to *f*_w_ of 95% (Figure 2b). Similar behavior was observed for compound **2**, while compounds **3** and **4** showed aggregation-caused emission quenching (Figure S2). Compounds **2** and **5** show TADF, but **3** and **4** do not. Generally, the low fluorescence quantum yields of organic compounds are explained by the low radiative rate constant *k*_r_ or a high nonradiative rate constant *k*_nr_.^5^ High *k*_nr_ values of organic compounds can be caused by intersystem crossing from excited singlet states to triplet states. However, in the case of compounds **2** and **5**, *k*_nr_ might decrease because of RISC from triplet states to the lowest singlet state: i.e., TADF. The enhanced emission intensity of compounds **2** and **5** in the solid state can most probably be attributed to their TADF properties.

To understand the nature of DF in more detail, PL and PH spectra of compound **5** doped in *m*CP were recorded (Figure 2c). The energies of the lowest close-placed singlet ^1^CT and triplet ^3^CT states were obtained, resulting in a small singlet–triplet splitting of 0.11 eV for compound **5**. In addition, the triplet LE state of acceptor thioxanthone (^3^LE_A_) at 2.85 eV was obtained. Closely placed ^1^CT, ^3^CT, and ^3^LE_A_ states are required for efficient TADF emitters.^31^Figure 2d demonstrates PL spectra of the film of compound **5** doped in *m*CP recorded at different excitation powers. Figure 2e shows a plot of the intensity of delayed fluorescence versus excitation power for the same film. A linear plot was obtained with a slope close to unity (Figure 2e). This observation confirms that the delayed fluorescence of compound **5** originates from the monomolecular process rather than from triplet–triplet annihilation (for which the slope is 2).^29,32^ Thus, the observed DF of compound **5** is TADF in nature. Similarly, the DF of compound **2** is also TADF in nature (Figures S3 and S4). In contrast, DF was not observed for compounds **3** and **4** dispersed in the host mCP or TCTA (Figure 1f). The main reason for such an observation are the greater values of Δ*E*_ST_ of compounds **3** and **4** than those of compounds **2** and **5** (Table 1). In addition, the wide energy gaps between ^3^CT and ^3^LE_A_ states of compounds **3** and **4** are not appropriate for TADF.

### 3.2 Electrochemical and Photoelectrical Properties

**Figure 3:**
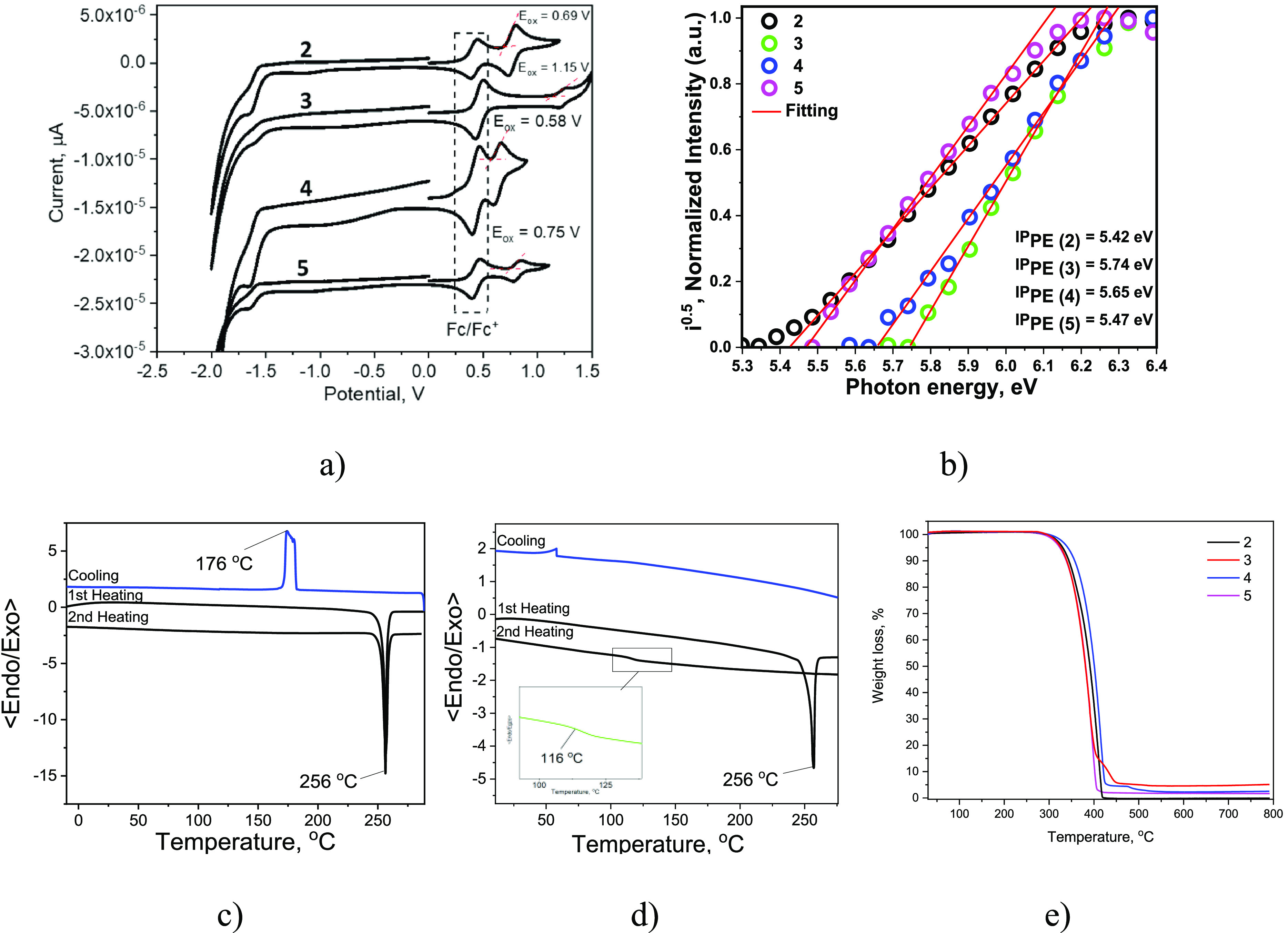
(a) Cyclic voltammograms of dilute solutions of compounds **2**–**5** in dichloromethane (sweep rate 100 mV/s). (b) Electron photoemission spectra of the solid samples of compounds **2**–**5**. DSC thermograms (c) of compound **2** and (d) of compound **3**. (e) TGA curves of compounds **2**–**5**.

The electrochemical properties of compounds **2**–**5** were investigated by cyclic voltammetry (CV). The data obtained are summarized in Table 2. Compounds **2**, **4**, and **5** showed reversible oxidation in the range of 0.69–1.15 V; meanwhile, compound **3** displayed quasi-reversible oxidation (1.15 V) (Figure 3a). The detected signals can be attributed to the oxidation of the donor moieties of the compounds. All of the compounds exhibited similar quasi-reversible reduction at potentials of around −1.50 V. It can be assigned to the reduction of the thioxanthone moiety. The ionization potentials of compounds **2**–**5** extracted from CV measurements (IP_CV_), i.e., obtained from the onset potential of their oxidation curves of compounds **2**–**5** were found to be of 5.49, 5.95, 5.38, and 5.55 eV, respectively. IP_CV_ values of compounds **2**, **4**, and **5** were found to be lower compared with that of compound **3**, indicating the stronger electron-donating ability of phenoxazinyl, 3,7-di-*tert*-butylphenothiazinyl, and 2,7-di-*tert*-butyl-9,9-dimethylacridanyl moieties compared to that of the 3,6-di-*tert*-butylcarbazolyl unit. Electron affinity (EA_CV_) values were determined according to the equation EA_CV_ = −(|IP_CV_| – *E*_g_^opt^) using the optical band gap energies taken from absorption spectra of the THF solutions of the compounds. The EA_CV_ values for compounds **2**–**5** were found to be 2.86, 3.10, 2.39, and 2.77 eV, respectively. Ionization potentials (IP_PE_) values of the thin films of the compounds coated on fluorine-doped tin oxide (FTO) were estimated by photoelectron emission spectrometry. The electron photoemission spectra of the compounds are shown in Figure 3b. The values are collected in Table 3. The IP_PE_ values of compounds **2**–**5** ranged from 5.42 to 5.74 eV. Electron affinity (EA_PE_) values for the solid samples of **2**–**5** were determined according to the equation EA_PE_ = IP_PE_ – *E*_g_^opt^ using the optical band gap energies taken from the absorption spectra of the films of the compounds. The values of IP_PE_ and EA_PE_ were used for the design of OLEDs.

**Table 2:** Thermal and Electrochemical Charge-Transporting Parameters of Compounds **2**–**5**

	compound			
	**2**	**3**	**4**	**5**
*T*_m_ (°C)^a^	256	256	205	242
*T*_cr_ (°C)	176			
*T*_g_ (°C)		116	105	104
*T*_–5%_ (°C)	330	325	342	323
*E*_onset_^ox^ vs Fc (V)^a^^b^	0.69	1.15	0.58	0.75
EA_CV_ (eV)^c^	2.86	3.10	2.39	2.77
IP_CV_ (eV)^d^	5.49	5.95	5.38	5.55
IP_PE_ (eV)^e^	5.42	5.74	5.65	5.47
*E*_g_^opt^ (eV)^f^	2.63	2.85	2.99	2.78
EA_PE_ (eV)^g^	2.79	2.89	2.66	2.69
μ_0h_/μ_0e_ (cm^2^ V^–1^ s^–1^)	2.3 × 10^–7^/–	1.6 × 10^–6^/1.7 × 10^–7^		5 × 10^–7^/6.7 × 10^–7^
β_h_/β_e_ (cm^1/2^ V^**–**1/2^)	11.5 × 10^–3^/–	6.2 × 10^–3^/8.5 × 10^–3^		6.9 × 10^–3^/3.2 × 10^–3^
μ_h_/μ_e_ (cm^2^ V^–1^ s^–1^)^h^	2.2 × 10^–4^/–	6.8 × 10^–5^/2.4 × 10^–5^		3.1 × 10^–5^/4.6 × 10^–6^

a *T*_m_, *T*_cr_, *T*_g_, and T_–5%_ are respectively melting temperature, crystallization temperature, glass-transition temperature determined by DSC (heating rate 10 °C/min, nitrogen atmosphere), *T*_–5%_ 5% mass loss temperature determined by TGA (heating rate 20 °C/min, nitrogen atmosphere).

b Onset of oxidation potential.

c EA_CV_ = −(|IP_CV_| – *E*_g_^opt^).

d IP_CV_ is 4.8 + *E*_onset_^ox^.^37^

e IP_PE_ is the ionization potential of compounds coated on FTO, with thin layers estimated by photoelectron emission spectrometry.

f *E*_g_^opt^ = 1240/λ_edge_, where λ_edge_ is the onset wavelength of the absorption spectrum of the nondoped solid film in the long wave direction.

g EA_PE_ = IP_PE_ – *E*_g_^opt^.

h μ_h_ or μ_e_ at 3.6 × 10^5^ V/cm.

**Table 3:** Characteristics of OLEDs

device	light emitting layer	*V*_on_^a^ (V)	max brightness^b^ (cd/m^2^)	PE_1000_/CE_1000_/EQE_1000_ (lm W^–1^/cd A^–1^/%)^c^	PE_max_/CE_max_/EQE_max_ (lm W^–1^/cd A^–1^/%)^d^	λ^e^ (nm)	CIE^f^
mCP-Doped OLEDs: ITO/MoO_3_/NPB/Light-Emitting Layer/TSPO1/TPBi/LiF:Al							
I	compound **2**	4.2	7121	6/10.9/3.2	6.7/11.2/3.4	544	(0.38, 0.55)
II	compound **3**	4.2	5185	2/3.8/1.8	2.5/4.3/2	482	(0.16, 0.3)
III	compound **4**	4.7	6789	1/2.1/1	1.1/2.2/1	557	(0.43, 0.53)
IV	compound **5**	4.1	13052	14.7/24/7.1	14.8/24.1/7.2	520	(0.27, 0.54)
TCTA-Doped OLEDs: ITO/MoO_3_/NPB/Light-Emitting Layer/TSPO1/TPBi/LiF:Al							
V	compound **2**	4.1	5683	5/10.5/4	5.7/10.6/4.1	565	(0.40, 0.51)
VI	compound **3**	4.6	3202	0.8/2.6/1.3	1.1/3.1/1.5	492	(0.22, 0.34)
VII	compound **4**	5.5	5203	0.8/2.4/0.9	1/2.5/0.9	543	(0.30, 0.43)
VIII	compound *5*	3.5	6703	10/18.7/6.4	21.2/29.3/10.3	544	(0.36, 0.54)

a Turn-on voltage at a luminance of 10 cd m^–1^.

b Maximum brightness.

c Power efficiency, current efficiency, and external quantum efficiency at 1000 cd m^–2^.

d Maximum power efficiency, maximum current efficiency, and maximum external quantum efficiency.

e Wavelengths of axima of EL spectra (λ_max_) at 6 V.

f CIE 1931 color coordinates.

### 3.3 Thermal Properties

The thermal transitions of the synthesized derivatives (**2**–**5**) were studied by thermogravimetric analysis (TGA) and differential scanning calorimetry (DSC) (Figure 3c-e and Figure S5). The data are given in Table 2. All of the compounds (**2**–**5**) were obtained as crystalline substances. The crystalline sample of **2** showed a melting signal at 256 °C in the first heating scan, and on cooling its crystallization was observed at 176 °C. The second heating scan revealed melting at 256 °C again. The melting temperatures of compounds **3**–**5** were observed at 256, 205, and 242 °C, respectively. As is shown in Figure 3d, the melted sample of compound **3** did not crystallize in the cooling scan and the second heating scan revealed a glass transition at 116 °C. Compounds **4** and **5** also formed molecular glasses after cooling their melts. Their glass transition temperatures were found to be 105 and 104 °C, respectively. The ability of compounds **3**–**5** to form molecular glasses can apparently be explained by the presence of *tert*-butyl groups attached to the donor moieties. Compound **3**, containing a rigid carbazole moiety, showed the highest glass transition temperature.

TGA experiments revealed 5% weight loss temperatures of compounds **2**–**5** of 330, 325, 342, and 323 °C, respectively. Single-stage TG curves up to complete weight loss of compounds **2**, **4**, and **5** show that 5% weight loss temperatures correspond to the temperatures of the onsets of sublimation of these compounds.

### 3.4 Charge-Transporting Properties

**Figure 4:**
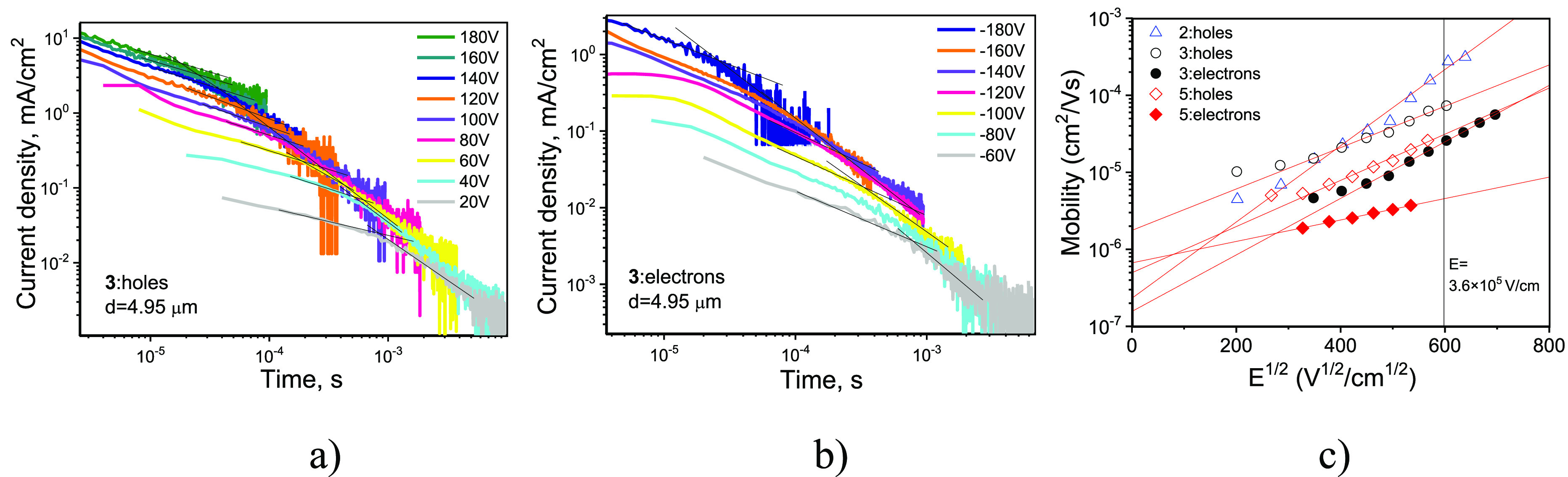
Hole (a) and electron (b) TOF signals for compound **3** and plots of charge carrier mobility versus electric field (c) for the layers of compounds **2**, **3**, and **5**.

The effect of different donor substituents of thioxanthones **2**–**5** of on their charge-transporting properties was investigated by the TOF technique.^33^ On determining the transit time (*t*_tr_) at the positive or negative voltages (*V*) applied to the optically transparent electrode ITO, drift mobilities of holes (μ_h_) or electrons (μ_e_), respectively, can be estimated using the formula μ_h,e_ = *d*^2^/(*Vt*_tr_) at a wide range of electric fields (*E*). In TOF measurements, a determination of *t*_tr_ is possible when the thickness (*d*) of the organic layer is greater than 1 μm.^34^ An interface generation of charges is required under a short laser excitation (355 nm, 6 ns). As can be seen in Figure 4a,b and Figure S6, *t*_tr_ was detectable from log–log plots of current transients for either holes (at positive voltages at ITO) or electrons (at negative voltages at ITO) for the planar capacitor-like samples ITO/vacuum-deposited organic layer/Al with thicknesses of 4.9 μm for **2**, 4.95 μm for **3**, 3.7 μm for **4**, and 5.6 μm for **5**. The thicknesses of layers of the studied compound were significantly larger than the inverse of their absorption coefficients (α) at the excitation wavelength (355 nm). Thus, *d* ≫ log 10/α. Charge injection blocking contacts were obtained at the electrode/organic layer interfaces. The work functions of electrodes (4.7 eV for ITO and 4.08 eV for Al) are very different from the ionization potentials and electron affinities of compounds **2**–**5** (Table 1). Nevertheless, current transients for holes and electrons were observed only for compounds **3** and **5** (Figure 4a,b and Figure S6). Current transients with *t*_tr_ for holes were obtained for compound **2.** Thus, bipolar charge transport was detected for **3** and **5** and hole transport was observed for **2.** Meanwhile, compound **4** did not show any charge transport (Figure 4a,b and Figure S6). The shapes of current transients show that compounds **2**–**5** are characterized by very dispersive charge transport. Apparently, because of the dispersity, charge transport was not detected for compound **4** (Figure S6). The dispersive charge transport of **2**–**5** could be attributed to their very twisted molecular structures and conformational disorder in the solid state.

Charge carrier mobilities versus electric fields are plotted in Figure 4c according to the Poole–Frenkel dependence (μ = μ_0_ exp β*E*^1/2^), where μ_0_ is the zero-field mobility and the β is Poole–Frenkel electric field dependence;^35^ the β values of compounds **2**–**5** are collected in Table 2. The values of μ_0_ and β were varied in the ranges of 1.7 × 10^–7^ to 1.6 × 10^–6^ cm^2^ V^–1^ s^–1^ and 3.2 × 10^–3^ to 11.5 × 10^–3^ cm^1/2^ V^–1/2^, respectively. At high electric field (e.g., at 3.6 × 10^5^ V/cm), compounds **3** and **5** showed higher hole mobilities than electron mobilities (Table 2). The charge-transporting properties of thioxanthones **2**–**5** are very sensitive to the donor substituent. This observation can apparently be explained by the different spatial frontier orbital distributions of compounds containing different electron-donating moieties (phenoxazine, 3,6-di-*tert*-butylcarbazole, 3,7-di-*tert-*butylphenothiazine, or 2,7-di-*tert*-butyl-9,9-dimethylacridane). In the layers of compounds **3** and **5**, which show bipolar charge transport, efficient hole and electron hopping between adjacent molecules occurs. It is caused by sufficient overlapping of both HOMO–HOMO and LUMO–LUMO molecular orbitals. In contrast, such overlapping is apparently not sufficient in the layers **2** and **4** probably because of the strong electron-donating abilities of phenoxazine and 3,7-di-*tert-*butylphenothiazine moieties. The highest hole mobility (3.1 × 10^–4^ cm^2^ V^–1^ s^–1^ at 4.1 × 10^5^ V/cm) was observed for phenoxazinyl-substituted thioxanthone (**2**). However, electron-transporting properties were not detected for this compound. Consequently, compounds **3** and **5** are more promising for applications as light-emitting materials of OLEDs, where both hole and electron transport are required.^36^

### 3.5 OLED Fabrication and Characterization

**Figure 5:**
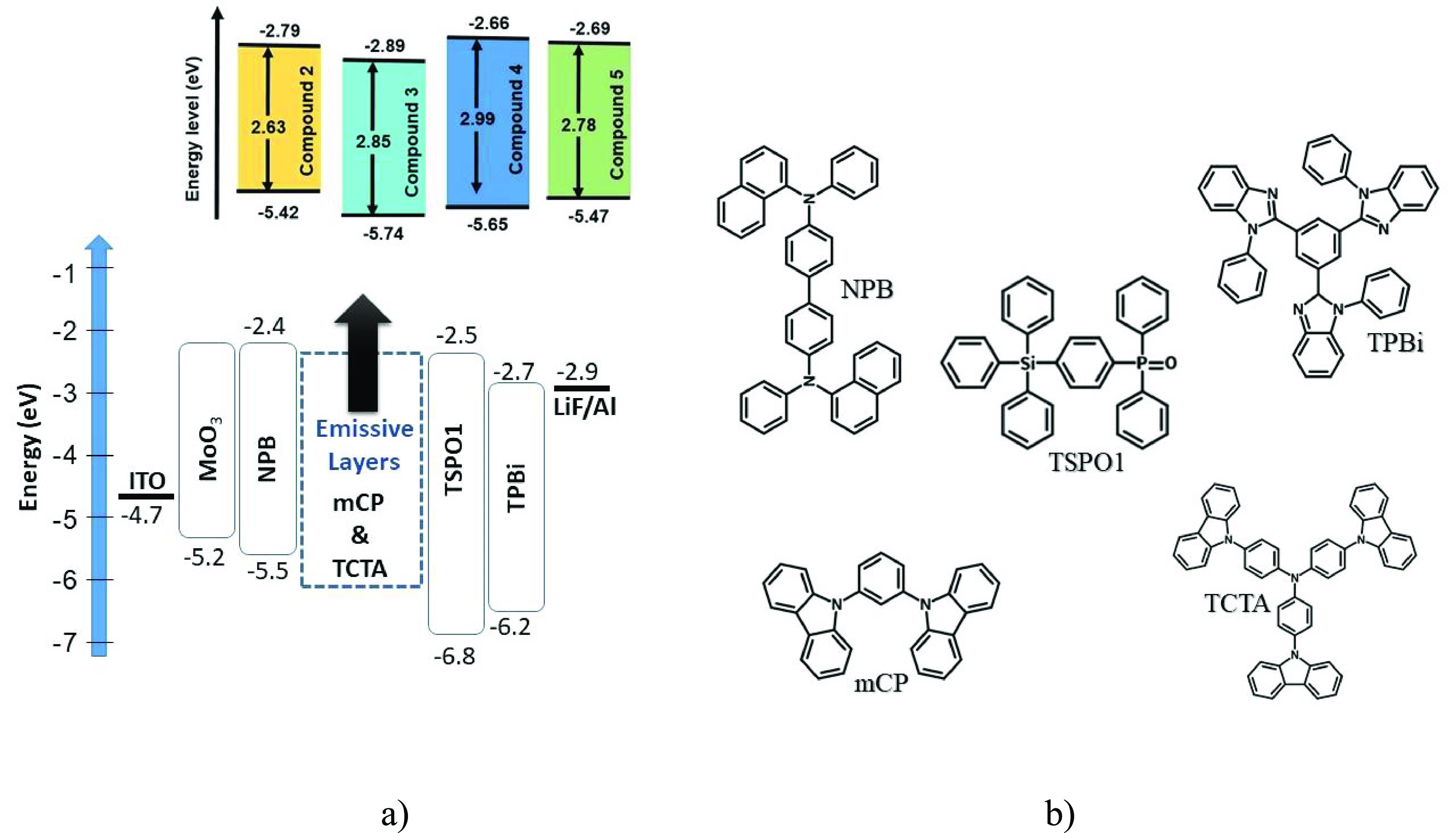
Equilibrium energy diagram (a) and the molecular structures (b) of the compounds used in the devices.

**Figure 6:**
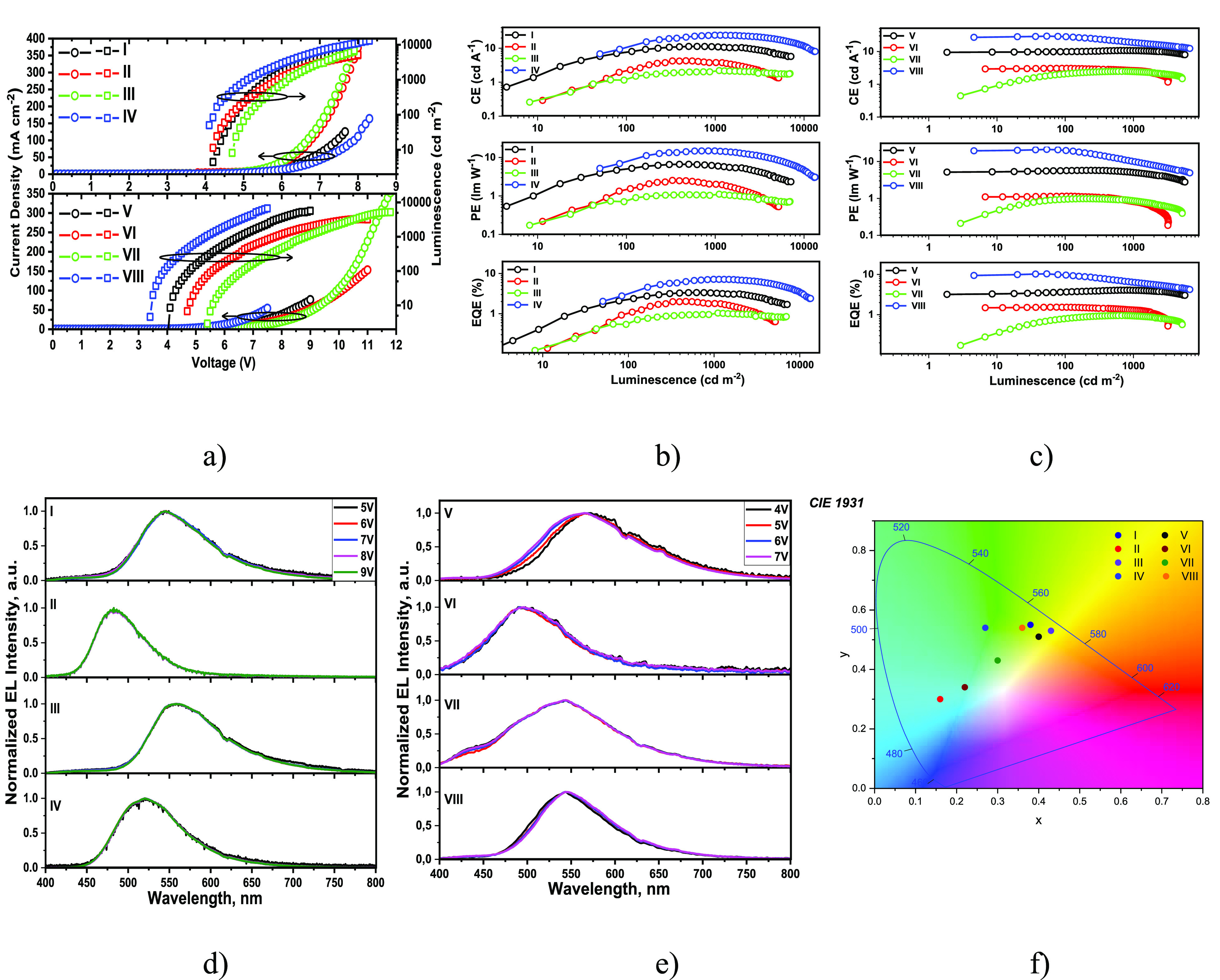
Current density and brightness versus voltage plots (a), current efficiency, power efficiency and external quantum efficiency versus luminescence plots of mCP (b)- and TCTA (c)-based devices, normalized electroluminescence spectra recorded under different applied voltages (d, e), and CIE 1931 color diagram (f).

The electroluminescent (EL) properties of compounds **2**–**5** were studied using the following structure of the devices: indium tin oxide (ITO), hole injecting layer of molybdenum oxide (MoO_3_) (0.5 nm)/hole-transporting layer of *N,N*′-di(1-naphthyl)-*N,N*′-diphenyl-(1,1′-biphenyl)-4,4′-diamine (NPB) (34 nm)/emissive layers of 10 wt % solid solutions of compounds **2**–**5** in the different hosts (20 nm)/hole blocking layer of diphenyl[4-(triphenylsilyl)phenyl]phosphine oxide (TSPO1)(6 nm)/electron-transporting layer of 2,2′,2″-(1,3,5-benzinetriyl)tris(1-phenyl-1-*H*-benzimidazole) (TPBi) (30 nm)/LiF (1 nm)/Al (80 nm). OLEDs containing the host *m*CP and compounds **2**–5 as emitters are denoted as devices I–IV, respectively. OLEDs containing TCTA as a host and compounds **2**–**5** as emitters are denoted as devices V–VIII, respectively. The equilibrium energy diagrams of devices I–VIII are schematically presented in Figure 5a. The energy levels IP_PE_ and EA_PE_ of the films of **2**–**5** were used for the design of the equilibrium energy diagrams (Table 2). According to the equilibrium energy diagrams of devices I–VIII, there are no energy barriers which may dramatically limit hole and electron injection and transport from the anode ITO and cathode LiF:Al to the light-emitting layer. As a result, devices I–IV were turned on at relatively low voltages of 4.09–4.69 V (Figure 6a and Table 3). Slightly lower turn-on voltages were obtained for devices V–VIII. This observation can be explained by the lower hole-injection barrier for TCTA-based devices than for *m*CP-based devices due to the shallower HOMO of TATA (−5.7 eV) in comparison to that of *m*CP (−5.9 eV). Reflecting the effect of the different donor substituents on optoelectronic properties of bipolar thioxanthones, the different electroluminescent colors and current (CE), power (PE), and external quantum efficiency (EQE) were obtained for devices I–IV (Figure 6b,c and Table 3). The maximum luminescence of all devices exceeded 5000 cd/m^2^ (Figure 6a and Table 3). Meanwhile, the maximum efficiencies were observed at a “useful” luminescence of 1000 cd/m^2^ due to the best hole–electron balance at high current densities.

Hole–electron pairs are expected to be efficiently formed within the light-emitting layers due to the presence of the hole- and electron-blocking interfaces light-emitting layer/TSPO1 and NPB/light-emitting layer, respectively. High triplet levels of NPB (2.38 eV)^30^ and TSPO1 (3.36 eV)^38^ prevent triplet leakage from the light-emitting layer to the neighboring functional layers. In addition, there are no signs of exciplex formation. These statements are confirmed by EL spectra resulting exclusively from emissions of **2**–**5** at different applied external voltages (Figure 6d,e).

The devices were characterized by blue, green, yellow, and orange EL with the corresponding commission internationale de l′Éclairage (CIE) chromaticity coordinates *x* and *y*, which are collected in Table 3 (Figure 6d–f). The wavelengths of emission peaks (λ_EL_) of 544, 482, 557, and 520 nm were observed for devices I–IV, respectively (Figure 6d and Table 3). The EL spectra of devices I–IV were similar to PL spectra of the films of the corresponding molecular mixtures of compounds **2**–**5** and *m*CP.

Device IV containing the derivative of thioxanthone and 2,7-di-*tert-*butyl-9,9-dimethylacridane (**5**) as an emitter exhibited a maximum current efficiency of 24.06 cd A^–1^, a maximum power efficiency of 14.84 lm W^–1^, and a maximum external quantum efficiency of 7.16%. Such efficiencies are in good agreement with high PLQY (50%) and TADF properties of compound **5** dispersed in the host mCP (Figure 1f and Table 1). The lowest EQEs were obtained for devices II and III. This observation is in good agreement with the absence of TADF properties of compounds **3** and **4** and relatively low PLQY values (Figure 1f and Table 1). Despite the similar PLQY values of compounds **3** and **4**, device II showed better efficiencies than device III. This observation can be explained by the different charge-transporting properties of compounds **3** and **4** (Figure 4c and Table 2).

The charge-transporting properties of the light-emitting layers could be further improved by selecting a more appropriate host. To support this presumption, devices V–VIII were fabricated. In the case of TCTA-based devices, the trend of electroluminescent properties of compounds **2**–**5** was practically the same as for the *m*CP-based devices. For example, the best maximum EQE of 10.3% was obtained for the device-VIII-based emitter **5**. This EQE value is higher than that observed for device IV (7.2%) most probably because of the improved charge-injecting/charge-transporting properties. It should be pointed out that phenothiazine as the donor moiety is present in the molecular structure of compound **4**. The formation of quasi-axial (q-ax) and quasi-equatorial (q-eq) phenothiazine conformers has been widely studied. It results in the multicolor changes of emission of phenothiazine-based TADF emitters under an external stimulus.^39,40^ Similarly to the interpretation given in previous works, we explain the shoulder peak at about 475 nm of PL and EL spectra of compound **4** dispersed in TCTA to the formation of q-ax and q-eq phenothiazine conformers.

Tests of optoelectronic properties of compounds **2**–**5** as emitters in OLEDs I–IV provide a good starting point for their selection as electroactive materials for many other devices. Their successful application as hole-transporting materials, hosts, or photosensitive materials may be predicted.

## 4 Conclusions

Organic semiconductors containing phenoxazinyl, 3,6-di-*tert*-butylcarbazolyl, 3,7-di-*tert*-butylphenothiazinyl, and 2,7-di-*tert*-butyl-9,9-dimethylacridanyl groups as electron donors and a thioxanthone moiety as an electron acceptor were synthesized and studied. Thermal, electrochemical, and photophysical properties of the compounds are discussed. Compounds containing 3,6-di-*tert*-butylcarbazolyl and 3,7-di-*tert*-butylphenothiazinyl moieties show aggregation-caused quenching, whereas compounds containing phenoxazinyl and 2,7-di-*tert*-butyl-9,9-dimethylacridanyl moieties show both solid-state luminescence enhancement and thermally activated delayed fluorescence. Compounds containing 3,6-di-*tert*-butylcarbazolyl and 2,7-di-*tert*-butyl-9,9-dimethylacridanyl moieties are characterized by bipolar charge transport, whereas a compound containing a phenoxazinyl moiety shows hole transport with a drift mobility reaching 3.1 × 10^–4^ cm^2^ V^–1^ s^–1^ at 4.1 × 10^5^ V/cm. The device with the light-emitting layer based on a thioxanthone derivative containing the 2,7-*tert*-butyl-9,9-dimethylacridane moiety exhibited a low turn-on voltage of 3.5 V and maximum current, power, and external quantum efficiencies of 29.3 cd/A, 21.2 lm/W, and 10.3%. Attachment of phenoxazinyl, 3,6-di-*tert*-butylcarbazolyl, 3,7-di-*tert*-butylphenothiazinyl, or 2,7-di-*tert*-butyl-9,9-dimethylacridanyl groups to the thioxanthone moiety allows obtaining compounds exhibiting green, blue, yellow, and sky blue electroluminescence, respectively.

## Supporting Information Available

^1^H, ^13^C, and MS spectra of synthesized compounds, DSC graphs of **4** and **5**, energies and transitions at S_1_ and S_2_ of compounds **2**–**5** calculated using TD-DFT at the B3LYP/6-31g(d,p) level, possible emission from S_1_ and/or S_2_ of compounds **2**–**5** in more polar solvents, TOF signals for holes and electrons of vacuum-deposited films of compounds **2**–**5**, additional photophysical data including PL spectra of the dispersions of compounds **2**–**5** in THF/water mixtures and PL, and phosphorescent spectra of the molecular mixtures of compounds **2**–**5** doped in *m*CP recorded at 77 K (PDF)
