# Supplementary material for: Effect of Substituents with the Different Electron-Donating Abilities on Optoelectronic Properties of Bipolar Thioxanthone Derivatives
Source: ACS Appl Electron Mater. 2023 Mar 28;5(4):2227–38. doi: 10.1021/acsaelm.3c00092 (PMC10134433; doi:10.1021/acsaelm.3c00092)
Supplement: Supplementary file 1 — el3c00092_si_001.pdf [file el3c00092_si_001.pdf]

Supporting Information

Effect of substituents with the different electron-donating abilities on optoelectronic properties of bipolar thioxanthone derivatives

Simas Macionis[1], Dalius Gudeika[1], Dmytro Volyniuk[1], Malek Mahmoudi[1], Jurate Simokaitiene[1], Viktorija Andruleviciene[1], Murad Najafov[1], Rita Sadzeviciene[2], Sigitas Stoncius[2], Juozas V. Grazulevicius[1,*]

[1]*Department of Polymer Chemistry and Technology, Kaunas University of Technology, K. Barsausko st. 59, LT-51423 Kaunas, Kaunas, Lithuania; *e-mail: juozas.grazulevicius@ktu.lt*

[2]*Department of Organic Chemistry, Center for Physical Sciences and Technology, Sauletekio Ave. 3, LT-10257 Vilnius, Lithuania*

2-(10H-fenoxazin-10-yl)-9H-thioxanthen-9-one ***(2)***

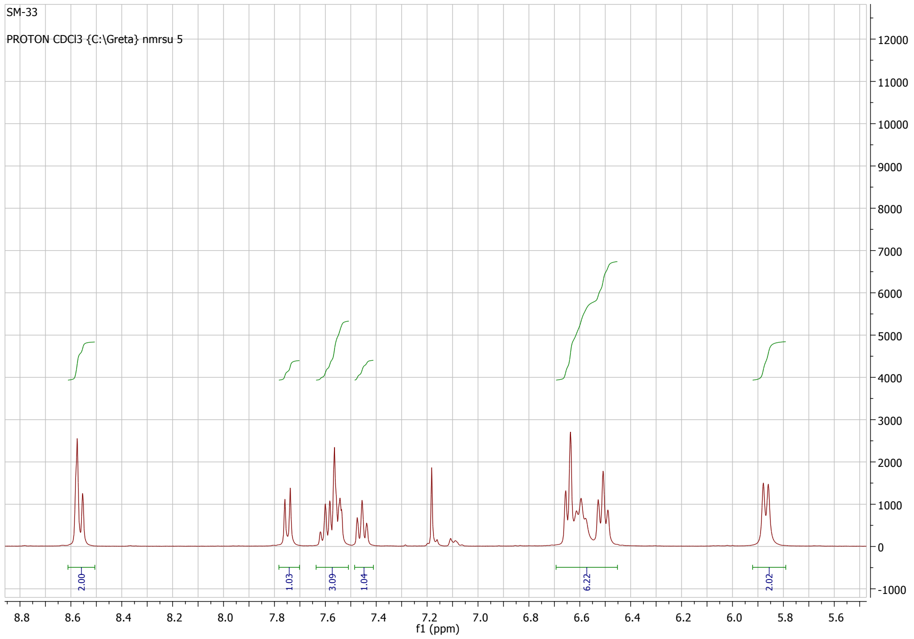

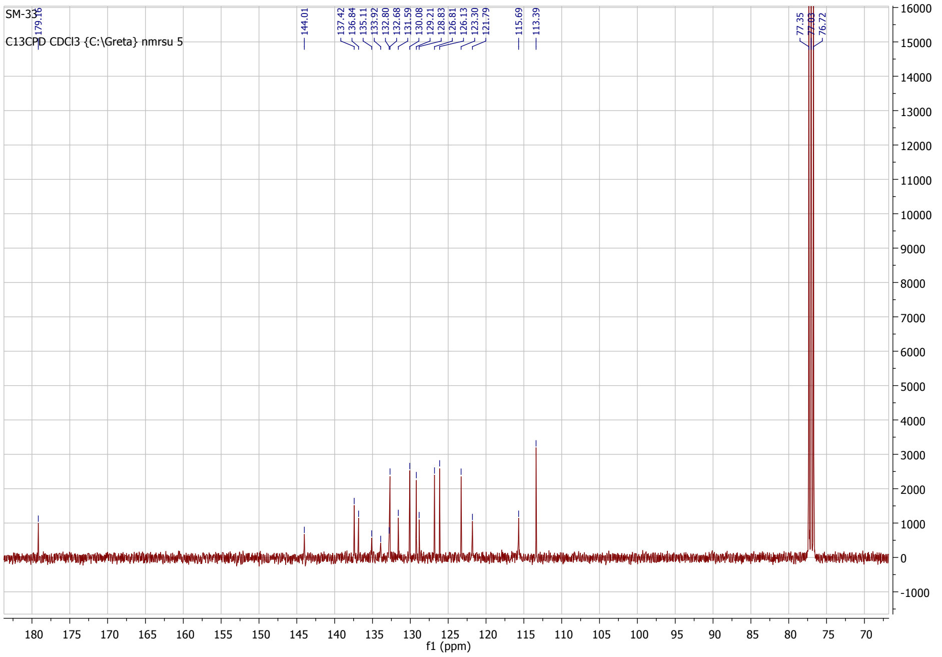
SM-33
C13CPD CDCl3 {C:\Greta} nmrsu 5
179.16
144.01
137.42
136.84
135.11
133.92
132.80
132.68
131.59
130.08
129.21
128.83
126.81
126.13
123.30
121.79
115.69
113.39
77.35
77.03
76.72
f1 (ppm)

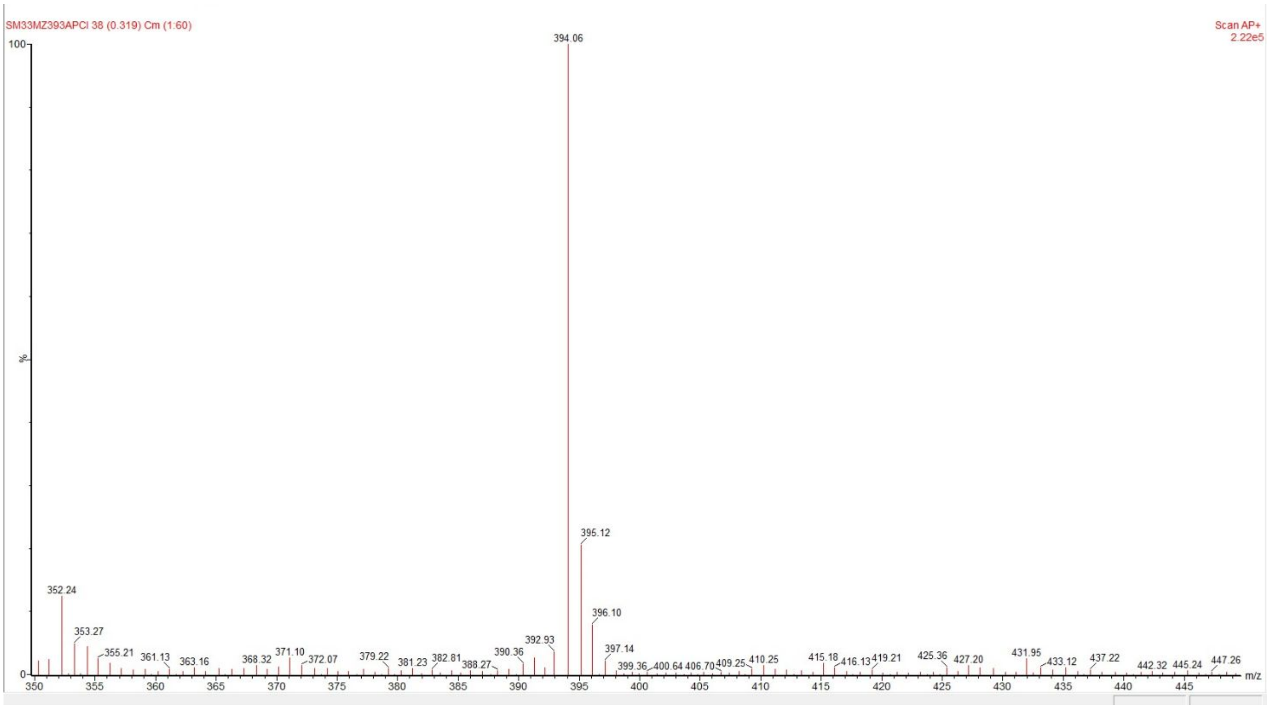
SM33MZ393APCI 38 (0.319) Cm (1:60)
Scan AP+
2.22e5
394.06
395.12
352.24
396.10
353.27
397.14
392.93
390.36
355.21
361.13
363.16
368.32
371.10
372.07
379.22
381.23
382.81
388.27
399.36
400.64
406.70
409.25
410.25
415.18
416.13
419.21
425.36
427.20
431.95
433.12
437.22
442.32
445.24
447.26
%
m/z

2-(3,6-Di-tert-butyl-9H-carbazol-9-yl)-9H-thioxanthen-9-one ***(3)***

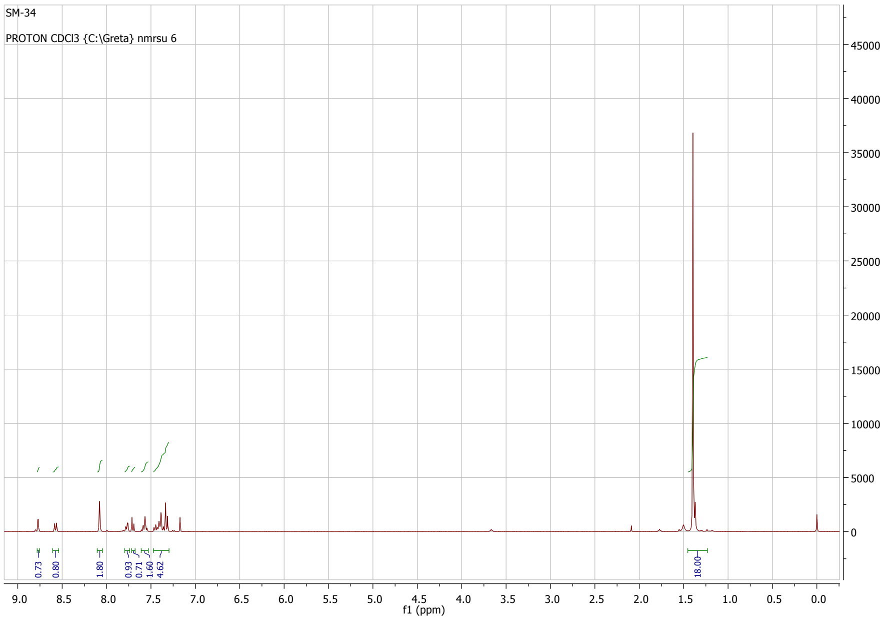

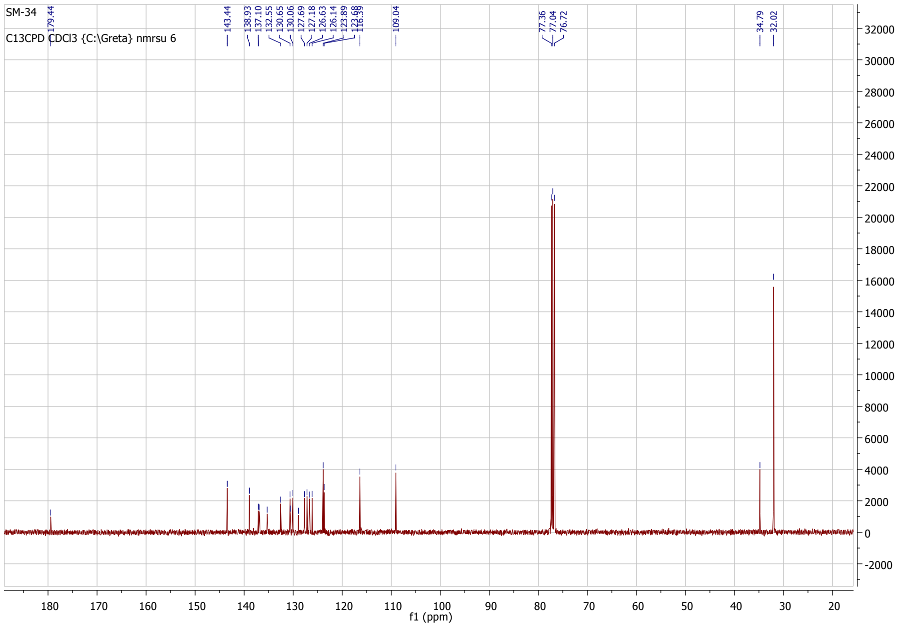

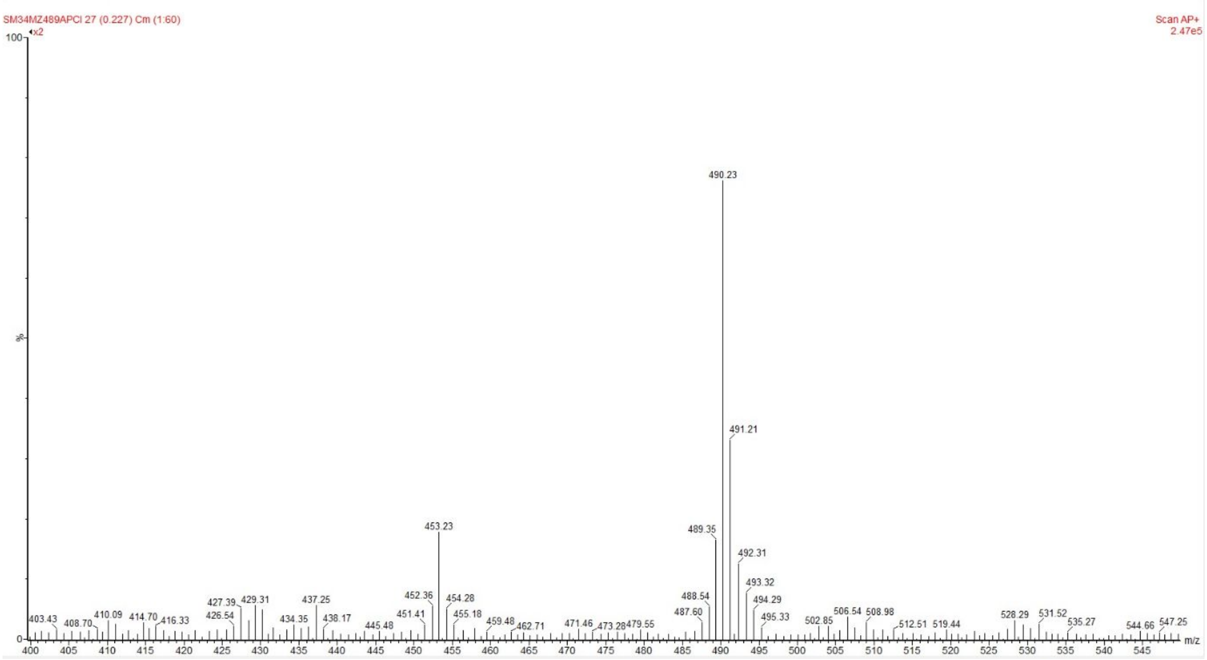

2-(3,7-Di-tert-butyl-10H-phenothiazin-10-yl)-9H-thioxanthen-9-one ***(4)***

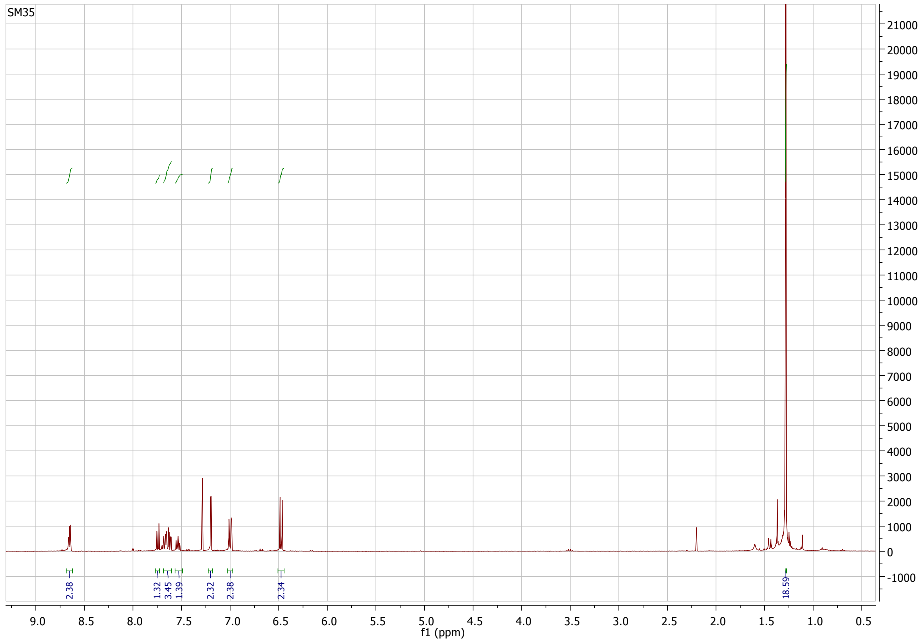

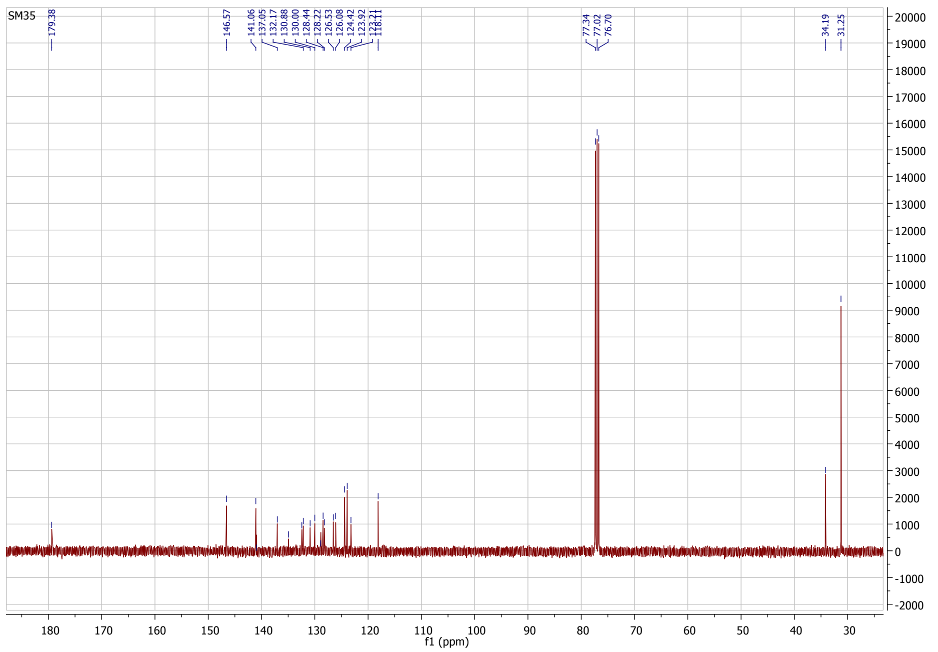
SM35
179.38
146.57
141.06
137.05
132.17
130.88
130.00
128.44
128.22
126.53
126.08
124.42
123.92
123.21
118.11
77.34
77.02
76.70
34.19
31.25
f1 (ppm)

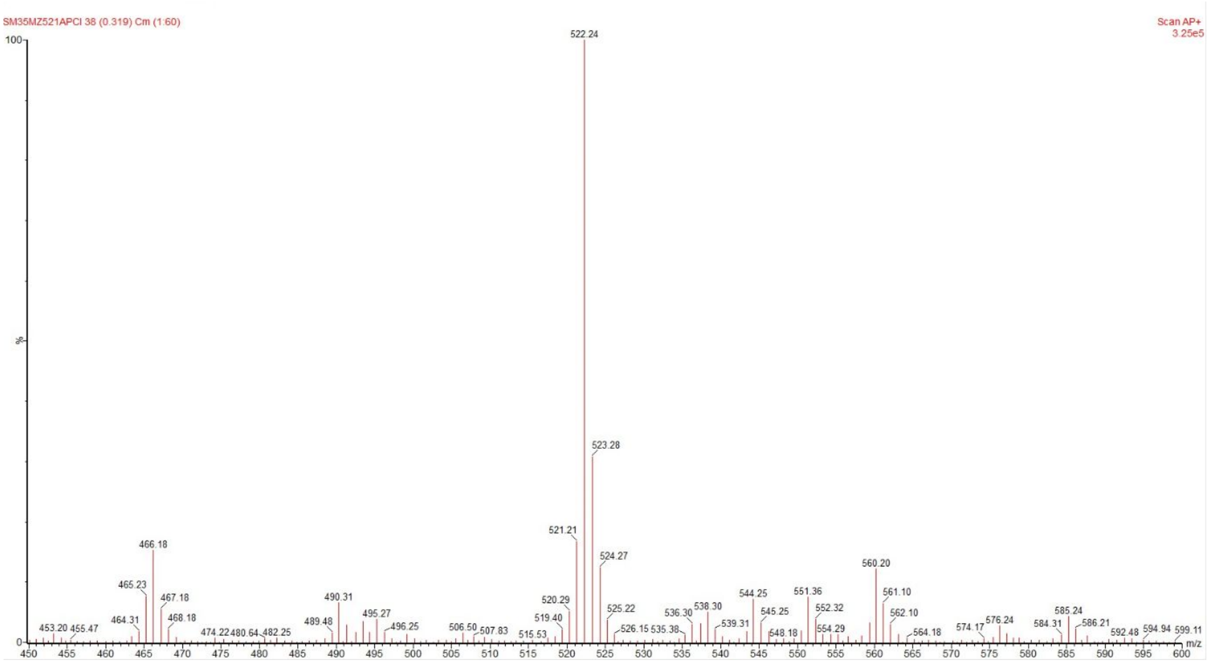
SM35MZ521APCI 38 (0.319) Cm (1:60)
Scan AP+
3.25e5
522.24
523.28
521.21
524.27
466.18
560.20
m/z

2-(2,7-Di-tert-butyl-9,9-dimethylacridin-10(9H)-yl)-9H-thioxanthen-9-one ***(5)***

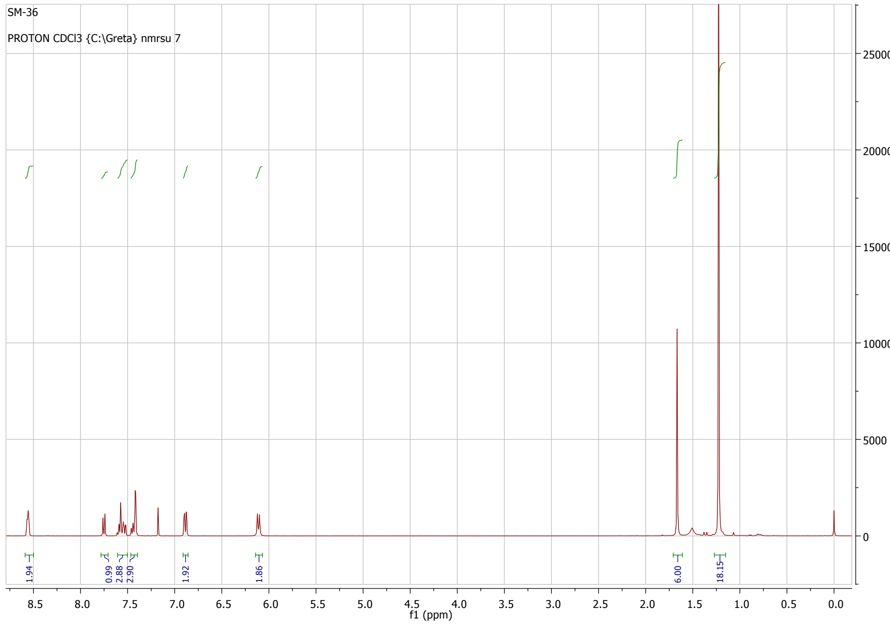

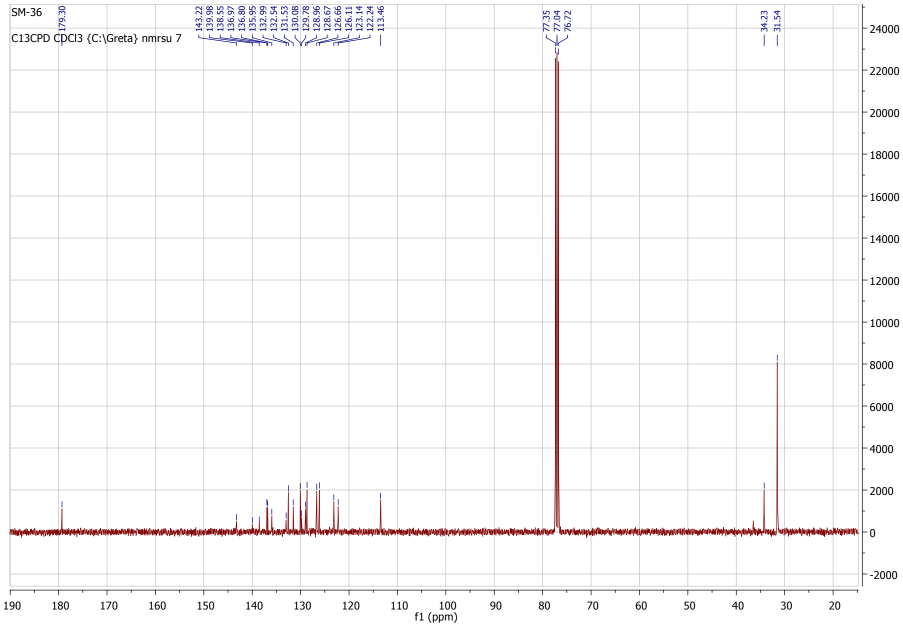

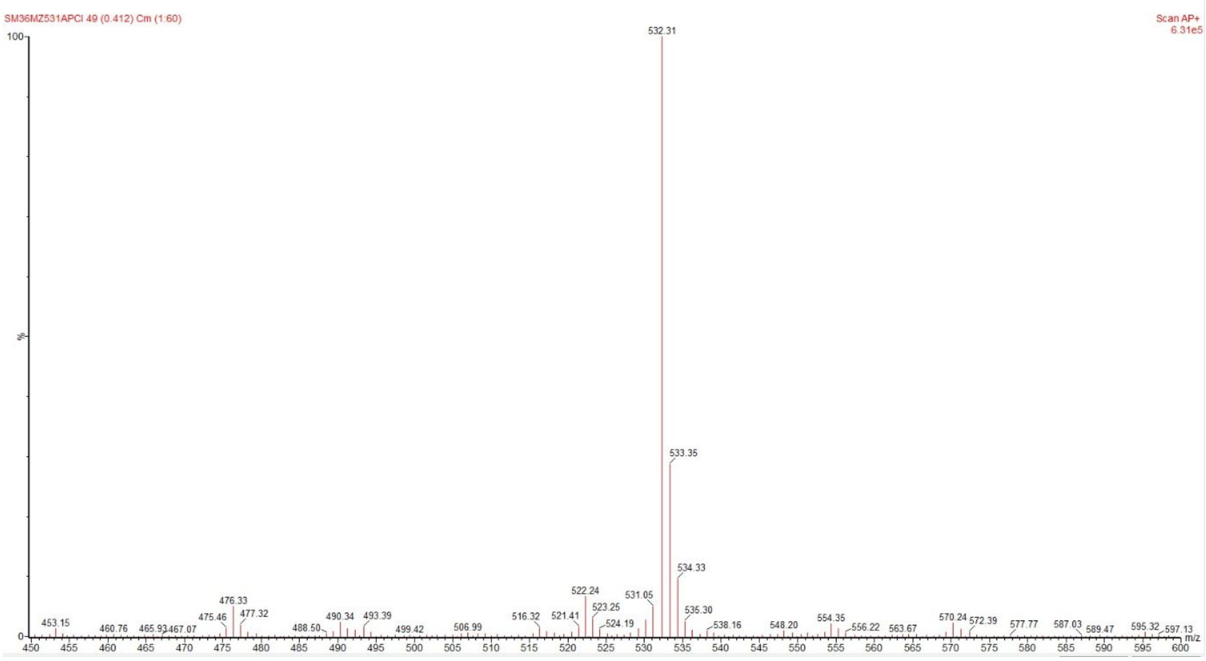
SM36MZ531APCI 49 (0.412) Cm (1:60)
Scan AP+
6.31e5
532.31
533.35
534.33
535.30
522.24
523.25
524.19
531.05
476.33
477.32
475.46
453.15
460.76
465.93
467.07
488.50
490.34
493.39
499.42
506.99
516.32
521.41
538.16
548.20
554.35
556.22
563.67
570.24
572.39
577.77
587.03
589.47
595.32
597.13
m/z
%

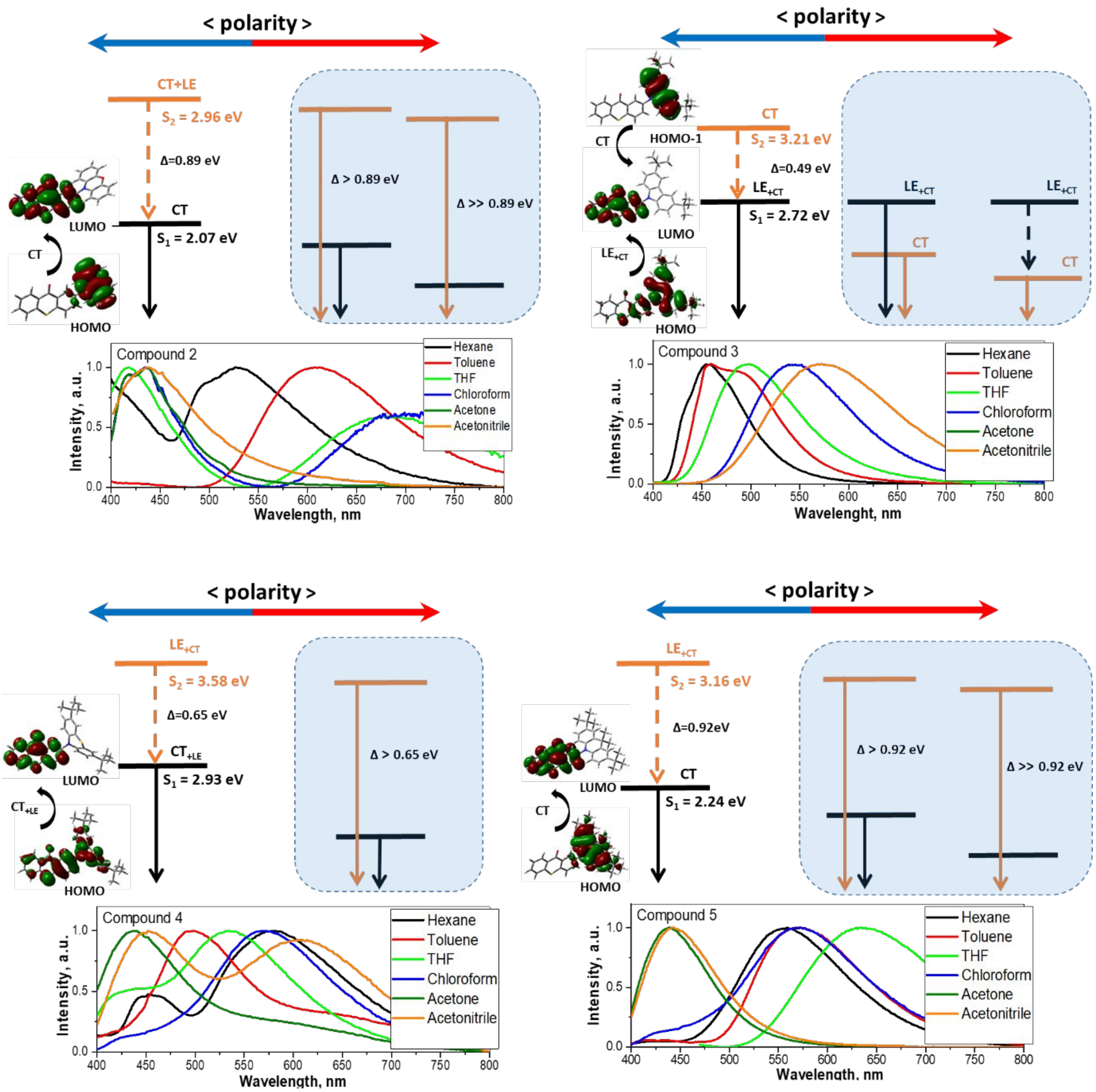

Figure S1. Energies and transitions from S_1 and S_2 of compounds **2**, **3**, **4**, and **5** calculated using TD-DFT at B3LYP/6-31g(d,p) level and corresponding PL spectra of the solutions (10^{-5} M) of compounds **2-5** (Figure 1b-e). The possible emission from S_1 and/or S_2 of compounds **2**, **3**, **4**, and **5** in more polar solvents is presented in the blue rectangle.

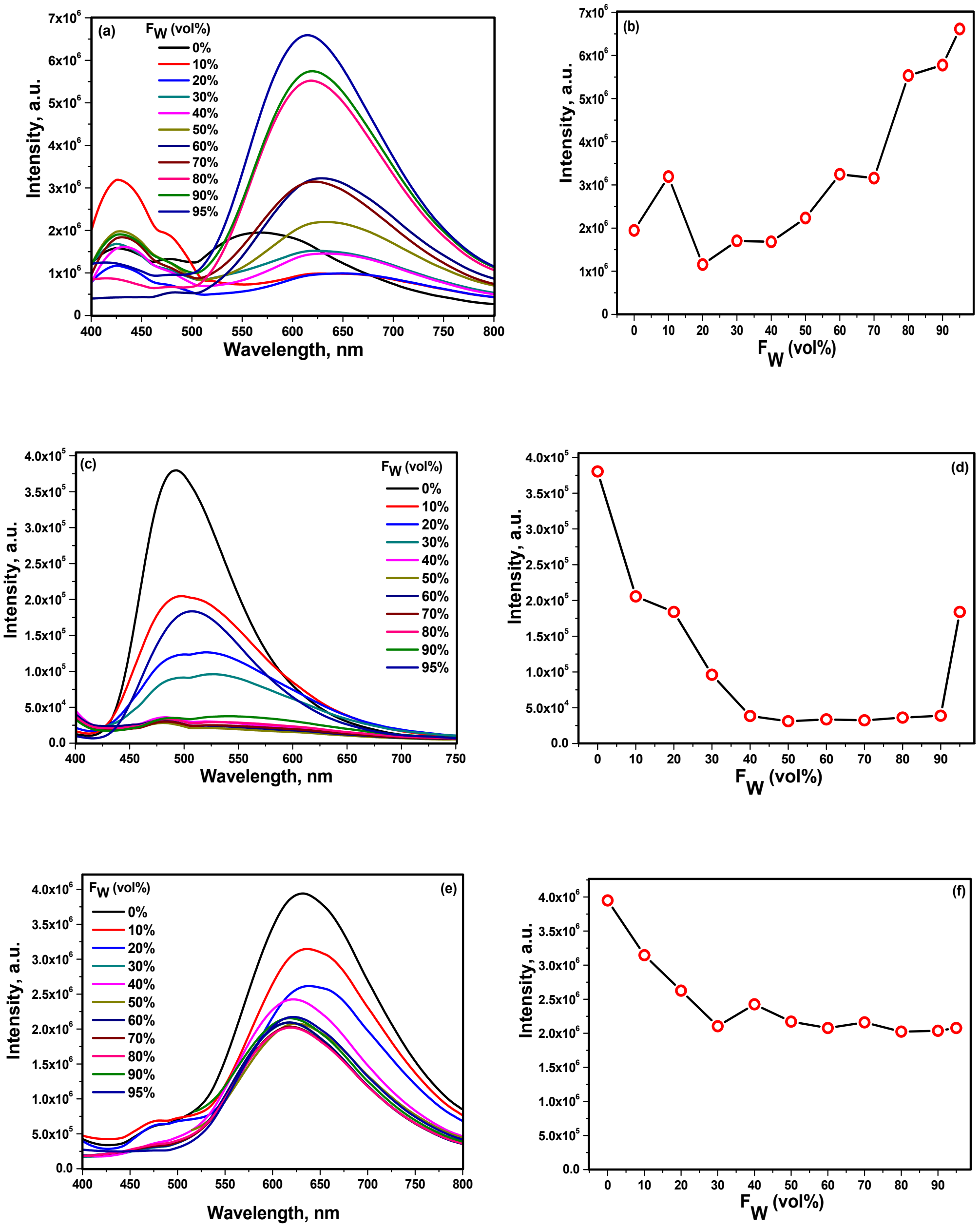

Figure S2. PL spectra of the dispersions of compounds (a) **2**, (c) **3** and (e) **4** in the THF/Water mixtures; the plots of PL maximum intensities versus water volume fraction in THF/Water mixtures for (b) **2**, (d) **3** and (f) **4**.

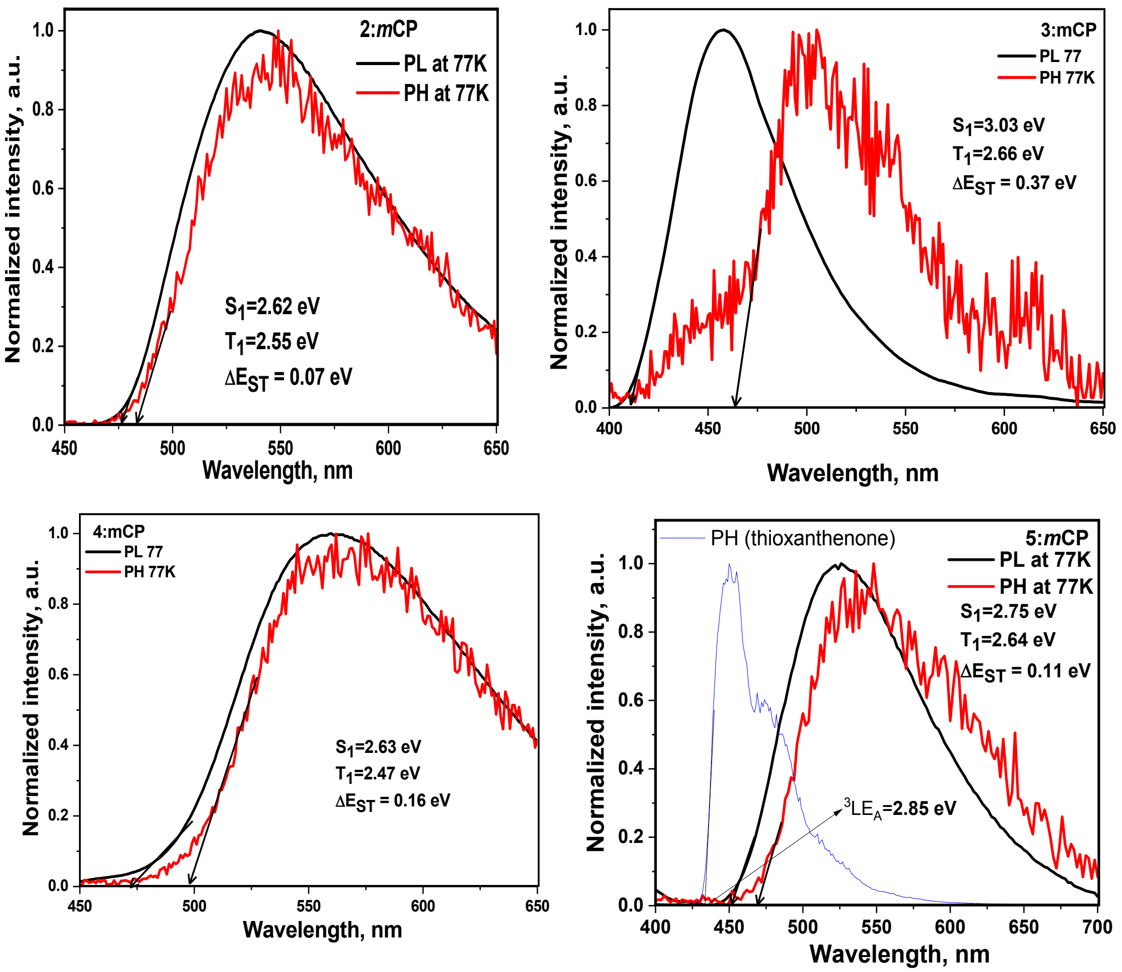

Figure S3. PL and PH spectra of the molecular mixtures of compounds **2-5** doped and *m*CP recorded at 77 K.

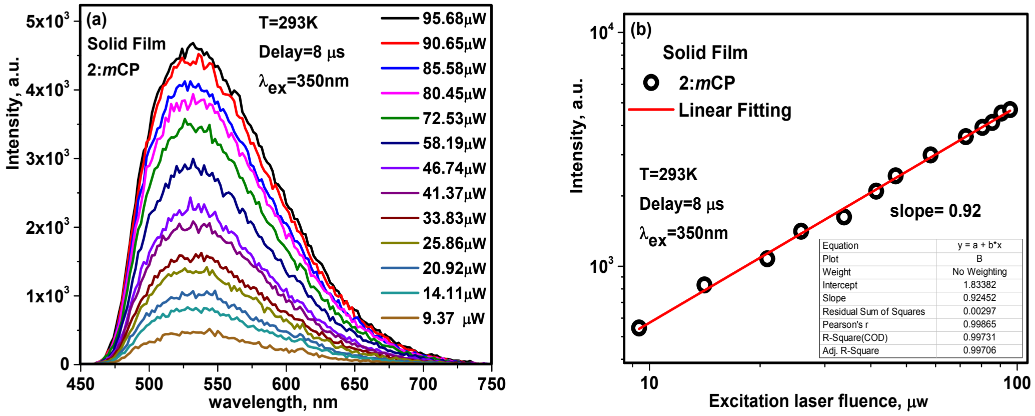

Figure S4. PL spectra (a) of the film of compound **2** doped in *m*CP recorded at the different excitation power. Delayed fluorescence intensity versus excitation power (b) of the film of compound **5** doped in *m*CP.

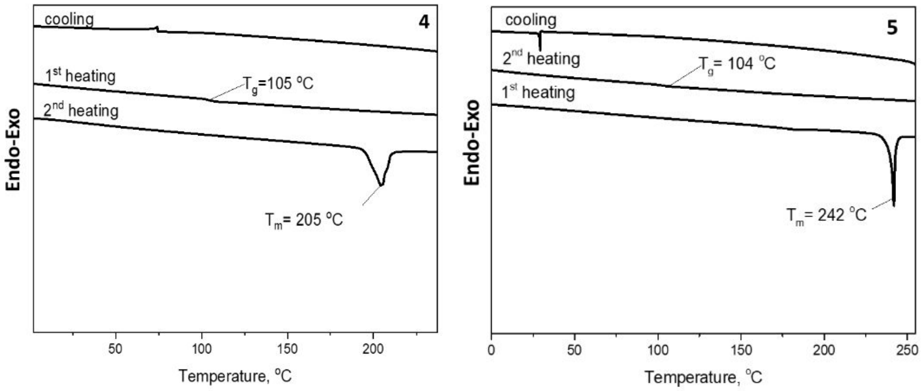

Figure S5. DSC curves of compounds **4** and **5**.

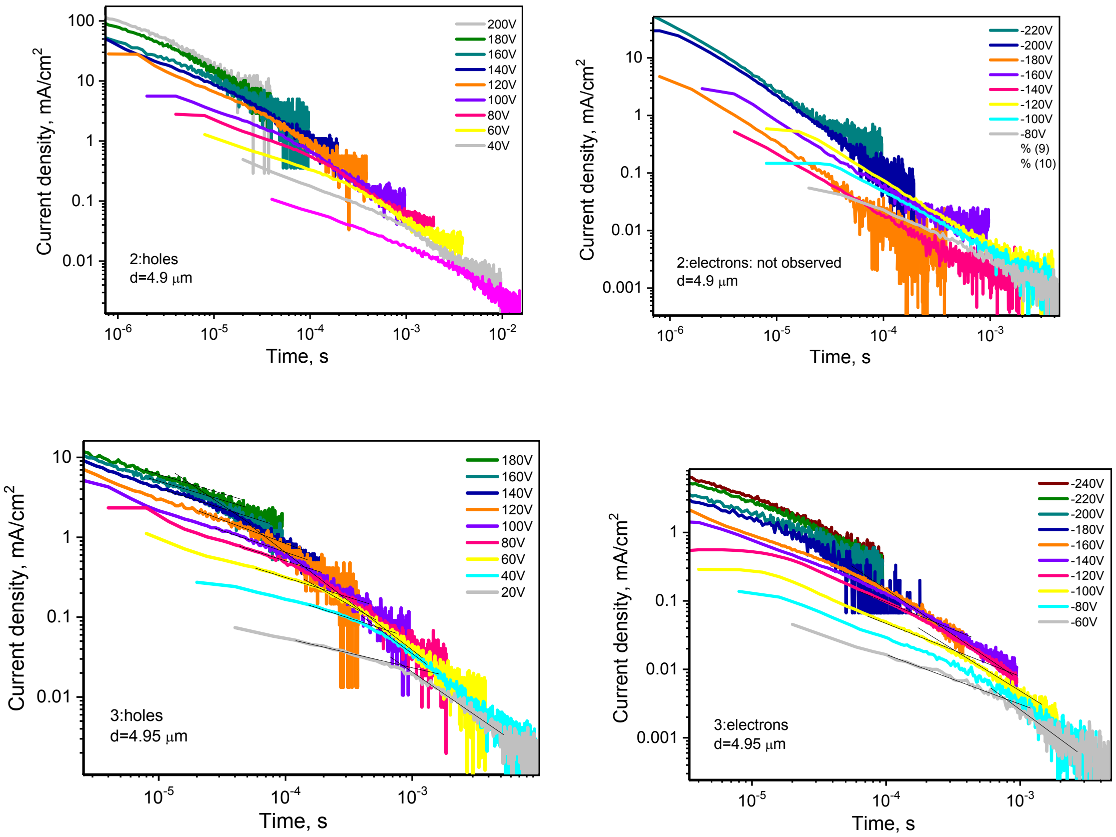

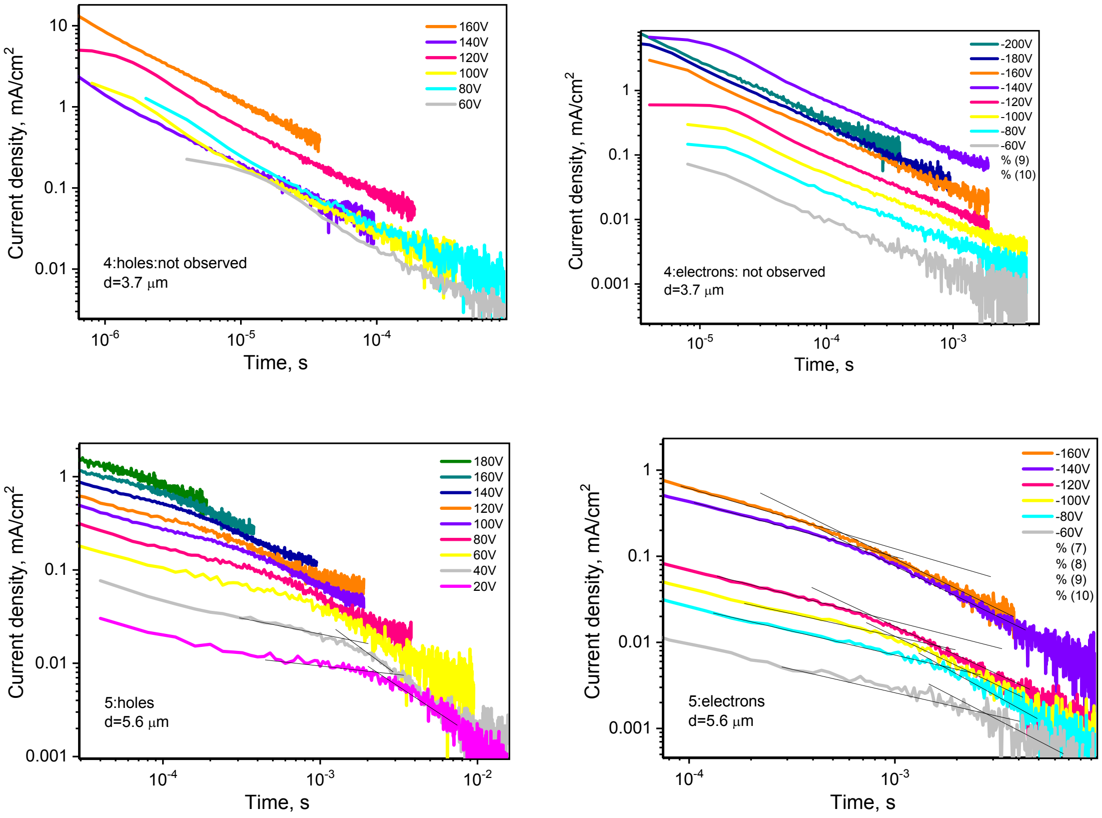

Figure S6. TOF signals for holes and electrons for vacuum-deposited films of compounds **2-5.**
